# Assessment of MRI to estimate metastatic dissemination risk and prometastatic effects of chemotherapy

**DOI:** 10.1038/s41523-022-00463-5

**Published:** 2022-09-02

**Authors:** George S. Karagiannis, Anthony Bianchi, Luis Rivera Sanchez, Kamal Ambadipudi, Min-Hui Cui, Jesus M. Anampa, Saeed Asiry, Yarong Wang, Allison S. Harney, Jessica M. Pastoriza, Yu Lin, Xiaoming Chen, Joan G. Jones, David Entenberg, Dana Haddad, Laura J. Hodges, Timothy Q. Duong, Joseph A. Sparano, Maja H. Oktay, Craig A. Branch, John S. Condeelis

**Affiliations:** 1 Department of Microbiology & Immunology, Albert Einstein College of Medicine, Bronx, NY USA; 2 Tumor Microenvironment and Metastasis Program, Albert Einstein Cancer Center, Bronx, NY USA; 3 Integrated Imaging Program, Albert Einstein College of Medicine, Bronx, NY USA; 4 Gruss-Lipper Biophotonics Center, Albert Einstein College of Medicine, Bronx, NY USA; 5 California State University, Bakersfield, CA USA; 6 Department of Anatomy and Structural Biology, Albert Einstein College of Medicine, Bronx, NY USA; 7 Department of Surgery, Montefiore Medical Center, Albert Einstein College of Medicine, Bronx, NY USA; 8 Department of Radiology, Montefiore Medical Center, Albert Einstein College of Medicine, Bronx, NY USA; 9 Gruss Magnetic Resonance Research Center, Albert Einstein College of Medicine, Bronx, NY USA; 10 Department of Medical Oncology, Montefiore Medical Center, Albert Einstein College of Medicine, Bronx, NY USA; 11 Department of Pathology, Montefiore Medical Center, Albert Einstein College of Medicine, Bronx, NY USA; 12 Department of Cell Biology, Albert Einstein College of Medicine, Bronx, NY USA; 13 Maediclinic Middle East, Department of Breast Imaging, Dubai, United Arab Emirates; 14 Department of Physiology and Biophysics, Albert Einstein College of Medicine, Bronx, NY USA

**Keywords:** Breast cancer, Cancer imaging, Metastasis

## Abstract

Metastatic dissemination in breast cancer is regulated by specialized intravasation sites called “tumor microenvironment of metastasis” (TMEM) doorways, composed of a tumor cell expressing the actin-regulatory protein Mena, a perivascular macrophage, and an endothelial cell, all in stable physical contact. High TMEM doorway number is associated with an increased risk of distant metastasis in human breast cancer and mouse models of breast carcinoma. Here, we developed a novel magnetic resonance imaging (MRI) methodology, called *TMEM Activity-MRI*, to detect TMEM-associated vascular openings that serve as the portal of entry for cancer cell intravasation and metastatic dissemination. We demonstrate that *TMEM Activity-MRI* correlates with primary tumor TMEM doorway counts in both breast cancer patients and mouse models, including MMTV-PyMT and patient-derived xenograft models. In addition, *TMEM Activity-MRI* is reduced in mouse models upon treatment with rebastinib, a specific and potent TMEM doorway inhibitor. *TMEM Activity-MRI* is an assay that specifically measures TMEM-associated vascular opening (TAVO) events in the tumor microenvironment, and as such, can be utilized in mechanistic studies investigating molecular pathways of cancer cell dissemination and metastasis. Finally, we demonstrate that *TMEM Activity-MRI* increases upon treatment with paclitaxel in mouse models, consistent with prior observations that chemotherapy enhances TMEM doorway assembly and activity in human breast cancer. Our findings suggest that *TMEM Activity-MRI* is a promising precision medicine tool for localized breast cancer that could be used as a non-invasive test to determine metastatic risk and serve as an intermediate pharmacodynamic biomarker to monitor therapeutic response to agents that block TMEM doorway-mediated dissemination.

## Introduction

Cancer cell dissemination occurs through specialized intravasation portals on blood vessels called Tumor Microenvironment of Metastasis (TMEM) doorways^1^. TMEM doorways consist of a perivascular macrophage, a tumor cell overexpressing the actin-regulatory protein Mammalian-enabled (MENA), and an endothelial cell, all in direct physical contact with each other^2–4^. Cancer cell intravasation at TMEM doorways occurs during tightly regulated transient localized vascular opening events triggered by the TMEM doorway macrophage, which expresses the angiopoietin receptor TIE2. Upon stimulation, TIE2^+^ macrophages at TMEM doorways secrete vascular endothelial growth factor-A (VEGFA), leading to the localized disruption of the underlying endothelial adherens and tight junctions and, as a consequence, the opening of the vessel wall (TMEM-associated vascular opening, or TAVO), localized vascular leakiness, and subsequent tumor cell transendothelial migration and intravasation^1^. As such, increased TMEM doorway density and activity in the primary tumor microenvironment have been associated with an increased incidence of circulating tumor cells (CTCs), disseminated tumor cells (DTCs) in the lungs (and other secondary sites), as well as metastases^1,5,6^. Consistent with these findings, TMEM doorway density in the primary tumor has been validated as an independent prognostic biomarker for distant recurrence in human breast cancer in three independent cohorts, including ~1150 patients^2,3,7^, and thus may serve as a biomarker for distinguishing potentially lethal from non-lethal cancers.

Consistent with these findings, TMEM doorway density in the primary tumor has been associated with distant recurrence in human breast cancer in three independent cohorts, including ~1150 patients^2,3,7^, and thus may serve as a biomarker for distinguishing potentially lethal from non-lethal cancers. In the first proof-of-concept study involving 30 case-control pairs of patients with and without distant recurrence, TMEM doorway density was significantly higher in patients with recurrence (*P* = 0.00006)^2^. In a subsequent prospective validation in 259 case-control pairs with and without distant recurrence from a population-based cohort, TMEM doorway density was likewise associated with an increased risk of distant metastasis in the subset of those with hormone receptor-positive, HER2-negative breast cancer (*P* trend = 0.004), but not in triple-negative or HER2-positive breast cancer. In the second prospective validation cohort, including 600 patients from a clinical trial cohort treated with adjuvant chemotherapy, proportional hazards models revealed a significant positive association between continuous TMEM doorway density score and early distant recurrence (*P* = 0.001) and locoregional plus distant recurrence (*P* = 0.00006) within 5 years of diagnosis in the subset of 297 patients with hormone receptor-positive, HER2-negative disease, but not in triple-negative or HER2-positive breast cancer. TMEM doorway density score correlated poorly with the 21-gene Recurrence Score (*r* = 0.29) and was significantly prognostic for early locoregional and distant recurrence (*p* = 0.05) in multivariate models including tumor size, grade, nodal metastasis, and the 21-gene Recurrence Score, with a trend toward first distant recurrence (*p* = 0.10). Finally, TMEM doorway density did not significantly correlate with tumor size or nodal status, and compared with hormone receptor-positive, HER2-negative disease, was significantly higher in triple-negative or HER2-positive breast cancer (*P* = 0.001 and *P* = 0.003, respectively), breast cancer subtypes associated with higher recurrence rate. The totality of the data, therefore, suggests a strong positive association between TMEM density in the primary tumor and breast cancer recurrence.

Assessment of cancer cell dissemination and metastasis in preclinical models is currently performed using histological endpoints, such as the number and localization of disseminated tumor cells at metastatic sites, as well as contextual changes in the tumor microenvironment. Although such methods provide some degree of morphological and molecular information, they are limited by the need to euthanize the animal and thus lack evidence regarding the dynamic nature of the phenomena they describe. Furthermore, only a small portion of the tumor is interrogated with the limited tissue sectioning typically used for the assessment of histological endpoints. The ability to observe cancer cell dissemination in situ, over short or long periods of time, and without the need for terminal procedures, has the potential to make a tremendous addition to our understanding of spatiotemporal changes in the tumor microenvironment, associated with cancer cell dissemination and metastasis. Indeed, such in vivo observations have been now made possible through the emergence of sophisticated imaging modalities in live animals, such as multiphoton intravital imaging^8–26^. Although these techniques offer great potential in basic research, they cannot be used clinically because they require genomic incorporation of artificially created fluorescent transgenes or direct injection of fluorescent reporters in the test subjects, and they also have poor depth of imaging in whole tissues. To circumvent this problem, we have investigated Magnetic Resonance Imaging (MRI) as an in vivo versatile and non-invasive imaging tool to simultaneously measure multiple tissue properties (e.g., structural, functional, and metabolic) associated with TMEM activity and metastatic dissemination in breast cancer.

In a clinical setting, contrast-enhanced MRI is commonly used for the detection and characterization of breast cancer. During a contrast-enhanced MRI exam, a Gadolinium-based contrast agent (GBCA) is typically injected, and the passage of GBCA into the tumor parenchyma is visualized by comparing image intensity changes pre- and post-contrast injection, also known as subtraction-based contrast^27^. Alternatively, high temporal resolution MRI of the dynamic passage of GBCA through tissue beds using longitudinal (T1) relaxation-based imaging can be used to measure the tissue’s permeability to the GBCA. Typically, GBCA affects MRI signal intensity in direct relation to its concentration within tissues, allowing for mathematical determination of its rate of exchange across the capillary. Endothelial permeability of GBCA can thus be determined directly from the observed arterial input and tissue responses of the MRI dynamic data. However, the typical approach for clinical assessment simply compares one or several post-GBCA images to a single pre-GBCA image, looking for features reflecting high contrast agent passage associated with tissue pathology^28^.

One method that has been employed to estimate permeability is k_trans_, which derives a bulk transfer constant (a rate constant) between intravascular and extravascular spaces from a combination of permeability and surface area^29^. Its estimation requires measurements of both the arterial input and the tissue response to the GBCA at a high temporal rate for several minutes. This method would allow measurement of TAVO by separating the extravascular transfer of GBCA that does not reflect typical permeability from the bolus efflux of plasma-borne GBCA into the tissue space. When many TMEM doorways exist within a tissue region, their opening is stochastic and temporally independent, which would appear in contrast-enhanced MRI as an overall increase in tissue permeability with individual TAVO events obscured. Thus, a dynamic measure of the tissue permeability, one which allows rates of efflux to be distinguished, may provide a means to detect TMEM doorways via this increased efflux rate. Further restriction to the initial time period post-GBCA administration may further enhance discrimination of TMEM doorway activity. We, therefore, implemented a ‘limited first pass’ assessment of the GBCA transfer rate between intravascular and extravascular spaces, calculated using only data from the initial bolus of the GBCA uptake, leading to the ability to select a range of transfer rates from the rate-histogram more likely to represent TMEM doorway activity, especially in highly permeable tissues^30^. In this study, we demonstrate that such a measure herein termed “TMEM Activity-MRI”, corresponds to the extent of TAVO events. We do not only demonstrate that TMEM Activity-MRI can be utilized in basic and translational settings to answer questions related to the molecular mechanism of cancer cell dissemination but also in the clinical setting as a newly proposed and non-invasive companion diagnostic for breast cancer patients.

## Results

### TMEM activity-MRI measurement correlates with TMEM doorway-associated vascular opening (TAVO) events

To measure TAVO events in tumors using magnetic resonance imaging (MRI), we developed an algorithm (Materials and Methods) to generate a dynamic contrast-enhanced first-pass deconvolution MRI map for mouse primary breast carcinomas. As discussed above, prior evidence indicates that tumor cell intravasation occurs exclusively in association with localized vascular openings at TMEM doorways^1^. To establish the TMEM Activity-MRI method, we utilized a previously established transgenic mouse model of spontaneous breast carcinoma, the Mouse Mammary Tumor Virus (MMTV) Polyoma Middle-T Antigen (PyMT) model (Supplementary fig. 1), also known as MMTV-PyMT (or simply PyMT), which recapitulates human breast cancer development and progression in a clinically relevant fashion^31–33^.

**Fig. 1:**
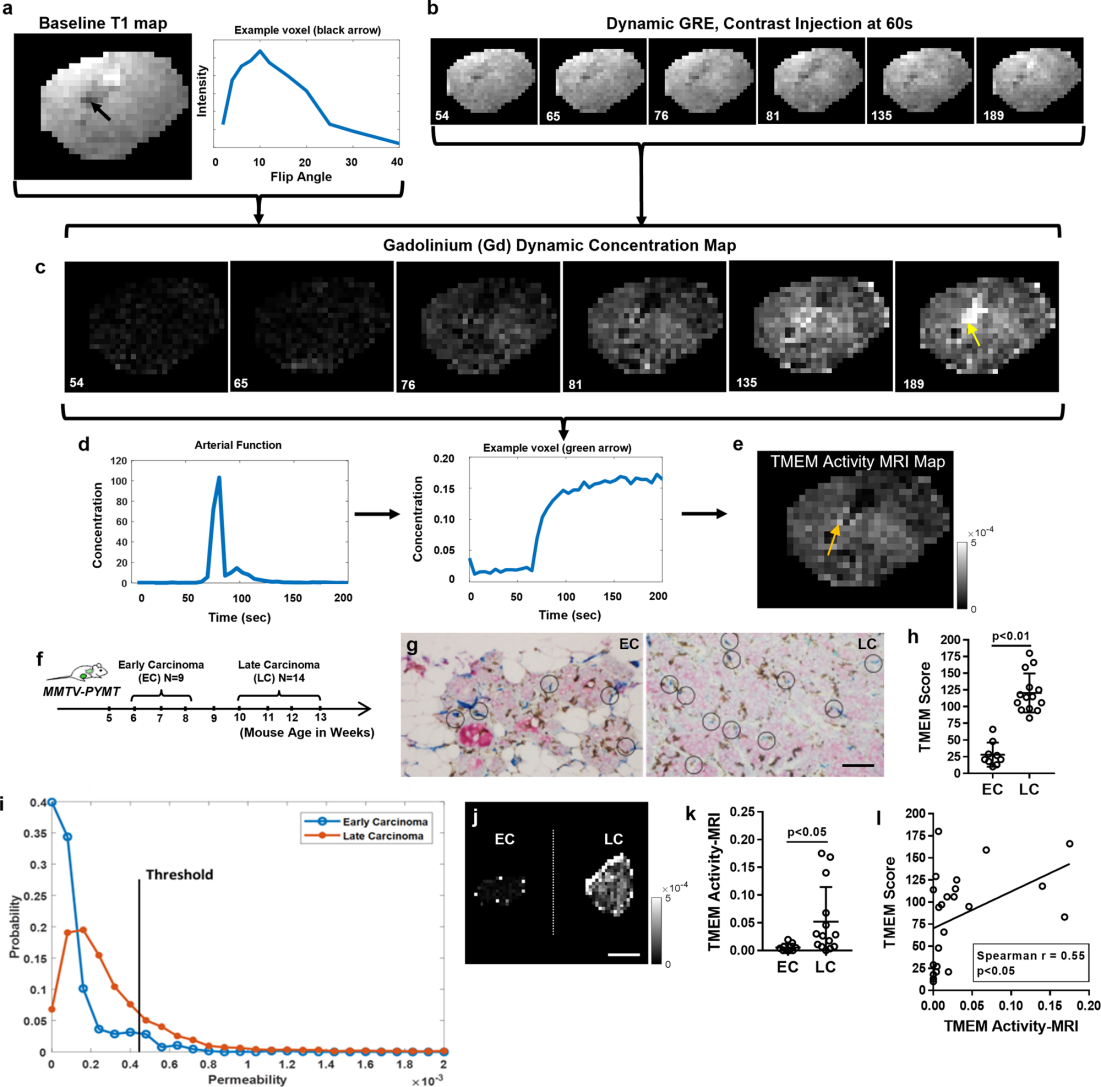
Development of TMEM activity-MRI assay. **a** T1 map of PyMT late carcinoma tumor (Left). A black arrow pointing at a potential blood/necrotic region. Example voxel intensity over varying flip angle (Right). This graph is fit to Eq. 5 at every voxel to calculate the baseline T1 map. **b** Dynamic GRE images over time post-contrast injection (at 60 s). Yellow arrow, a small amount of visual change over time. **c** Gadolinium-based contrast agent (GBCA) concentration map over time, calculated using the baseline T1 map and dynamic GRE using Eq. 8. Yellow arrow, very slow leak area and most likely necrotic, showing up as small in the K_fp_ image, indicating necrotic regions may have low K_fp_ values. **d** GBCA concentration of the arterial source (left), where the first pass of the contrast agent is the first peak, and the second smaller peak suggests GBCA recirculation. An example voxel from within the tumor with fast uptake of contrast agent (right). Note the scale difference with the arterial source. **e** Final TMEM Activity-MRI map calculated using Eq. 4. Orange arrow, hyper-intense voxel, i.e., larger value in K_fp_ is indicative of increased TAVO events. **f** Experimental design and mouse cohort composition. MMTV mouse mammary tumor virus, PyMT Polyoma Middle-T antigen, EC early carcinoma, LC late carcinoma. **g** TMEM identification by triple-stain immunohistochemistry (IHC) and representative images from early (EC) and late (LC) carcinoma MMTV-PyMT mice. Scale = 100um **h** Quantification of TMEM doorways (TMEM doorway score), assessed in 10 high-power fields (HPFs) in mice shown in (**g**). Mann–Whitney *U*-test. **I** Frequency histogram of the combined Early (EC) and Late (LC) Carcinoma mouse cohorts. The threshold is representative of creating the TMEM Activity-MRI Eq. 9. The optimal threshold was calculated to be ~0.001. **j** Representative TMEM Activity-MRI maps of mouse tumors by magnetic resonance imaging (MRI) from early (EC) and late (LC) carcinoma MMTV-PyMT mice. Hyper-intense voxels correspond to TMEM hotspots (i.e., increased number of TAVO events). Scale = 1 mm. **k** Quantification of TAVO events (a.k.a. TMEM activity), assessed via the TMEM Activity-MRI assay, in mice shown in (**j**). Mann–Whitney *U*-test. **l** Correlation between TMEM score (as quantified in **h**) and TMEM Activity-MRI (as quantified in **k**) in early and late carcinomas of the MMTV-PyMT mice shown in (**j**). Spearman’s rank correlation coefficient. Error bars: standard deviation (SD).

MMTV-PyMT mice bearing spontaneous breast tumors were subjected to a dynamic MRI protocol using a 9.4 T 31 cm Agilent Direct Drive imaging system. A baseline T1 map was acquired using the variable flip angle (FA) approach and gradient recalled echo (GRE) imaging (Fig. 1a), similar to previous publications^34,35^, with slight modifications as described in Materials and Methods. To delineate regions of interest (ROIs) that correspond to the tumor mass, the GRE image with a FA of 12 is used as it delineates the tumor tissue versus the normal tissue with the highest contrast compared to the other images, and it facilitates the anatomical demarcation of the tumor margins (Supplementary Fig. 2a–d). A dynamic GRE sequence is initiated, and the gadolinium-based contrast agent (GBCA) is injected after 60s using a standard dose of 140 μL of 0.1 mmol/Kg gadopentetate dimeglumine at a rate of 20 µL/sec (Fig. 1b). Using the baseline T1 map and the dynamic GRE sequence, a GBCA dynamic concentration map is then calculated from the relaxivity map, as previously reported^30^, giving an estimate of GBCA concentration for each individual voxel (Fig. 1c). From the dynamic concentration image series, the arterial source is identified (Fig. 1d; left panel), and an arterial input concentration curve is subsequently determined. An example region of the tumor with fast uptake is shown in the pipeline (Fig. 1d; right panel). Importantly, finding the arterial source also allows for the normalization among different subjects (Materials and Methods). As a next step, the time series of each voxel is fit to equation #4 (Materials and Methods) to generate the corresponding TMEM Activity-MRI map (Fig. 1e). Importantly, high TMEM doorway activity is reflected by voxels with hyperintensities in the TMEM Activity-MRI map (Fig. 1e; orange arrow) due to large endothelial openings. Voxels with low intensity in the TMEM Activity-MRI map likely reflect inactive TMEM doorways or regions devoid of TMEM doorways. Necrotic or non-perfused regions appear as either hyper-intense or hypo-intense voxels on T1W images, depending on whether fast- (open vasculature, Fig. 1a; black arrow) or slow-contrast-uptake (avascular) regions (Fig. 1c, yellow arrow) are present respectively, thus both yielding little or no permeability in the first-pass GBCA uptake (i.e., TMEM Activity-MRI) maps (Fig. 1e).

MMTV-PyMT mice, which spontaneously develop breast carcinoma, have been shown to form an increasing number of active TMEM doorways throughout tumor progression^1^. Therefore, a comparison between early (EC) and late (LC) carcinomas not only represents an excellent way to study TAVO events and cancer cell dissemination but also an excellent model to develop a sensitive MRI measurement corresponding to TMEM-dependent contrast agent leakage, as described in the pipeline above (Fig. 1a–e). To this end, we first generated an MMTV-PyMT mouse cohort (Fig. 1f), which included mice belonging to age groups corresponding to early carcinoma (EC; 6–8-week old, *N* = 9) and late carcinoma (LC; 10–13-week old, *N* = 14), as described previously^32^. The individual mouse tumors were also examined histologically in a retrospective manner (i.e., upon tumor resection following an MRI session) to confirm early- or late-stage carcinoma status by histopathology (Supplementary Fig. 1). As expected and also reported previously^1,36^, TMEM doorway assembly was significantly (*p* < 0.01; Mann–Whitney *U*-test) increased in LC compared to EC samples (Fig. 1g, h and Supplementary Fig. 3a). Because TMEM activity is increased in late-stage compared to early-stage PyMT carcinomas, we anticipated that such differences should be reflected in the TMEM Activity-MRI maps, as developed above (Fig. 1a–e). To look into this possibility, all PyMT breast tumor images were manually segmented from the TMEM Activity-MRI images, and their corresponding histograms were pooled for each group together and graphed (Fig. 1i). In this analysis, *k*_fp_ rates less than 0 and above 40 × 10^−3^ were masked off as error voxels (refer to Materials and Methods). This histogram analysis suggested that late carcinomas had a lower frequency of hypo-intense voxels (0.0–0.001) but a higher frequency of hyper-intense voxels (0.001–0.04) compared to early carcinomas (Fig. 1i), which was consistent with our hypothesis of an expected pattern of increased TMEM doorway-dependent vascular opening during breast cancer progression.

To establish easily interpretable and biologically relevant endpoints for statistical comparisons, we next calculated an MRI feature by combining the histogram analysis (Fig. 1i) from the TMEM Activity-MRI maps with permeability thresholding (Fig. 1i; black line). The calculated MRI measurement, simply termed “TMEM Activity-MRI,” is calculated from the corresponding TMEM Activity-MRI map and represents the ratio of the number of tumor voxels presenting with a permeability score above a certain threshold divided by the number of total voxels within the tumor ROI. As such, TMEM Activity-MRI signifies a suitable metric for specifically assessing the tumor compartment that contains the leakiest blood vessels in the entire tumor. In addition, TMEM Activity-MRI avoids potential bias coming from tumor size variations since it gives the percentage, and not the absolute value, of the voxels in a given tumor that has the highest permeability. Upon quantification, it became evident that TMEM Activity-MRI was significantly (*p* < 0.05; Mann–Whitney *U*-test) higher in the LC compared to the EC cohort in MMTV-PyMT mice (Fig. 1j, k). In support of this, TMEM Activity-MRI significantly (*p* < 0.05; Spearman rho = 0.55; Spearman’s rank correlation) correlates with TMEM doorway score (Fig. 1l), suggesting a positive correlation between TMEM Activity-MRI and tumor progression-dependent increases of TMEM doorway number. Taken together, observations shown in Fig. 1 suggest that the newly developed TMEM Activity-MRI measurement can successfully capture changes in TMEM doorway score, for instance, the increase in TMEM doorway number during the progression from early- to late-stage carcinoma) in a (patho)physiologically relevant preclinical model of mammary carcinoma.

### Clodronate-mediated reduction of TMEM doorways decreases TMEM Activity-MRI

**Fig. 2:**
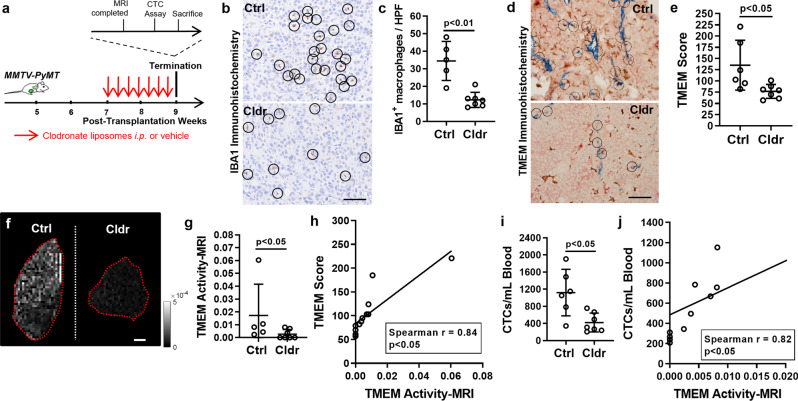
TMEM activity-MRI is suppressed, as a result of reduced TMEM doorway formation, using clodronate-mediated macrophage depletion. **a** Experimental strategy and mouse cohort composition for MMTV-PyMT mice subjected to clodronate-dependent macrophage depletion. PyMT Polyoma Middle-T antigen, CTC circulating tumor cell. **b** Identification of macrophages by IBA1 immunohistochemistry and representative images from MMTV-PyMT mice, treated with either control or clodronate liposomes. Scale = 100 um. **c** Quantification of IBA1^+^ macrophages, as averaged in 10 high-power fields (HPFs) in MMTV-PyMT mice shown in (**b**). Mann–Whitney *U*-test. **d** TMEM identification by triple-stain immunohistochemistry (IHC) and representative images from MMTV-PyMT mice, treated with either control or clodronate liposomes. Scale = 100 um. **e** Quantification of TMEM doorways (TMEM score), as assessed in 10 high-power fields (HPFs), in MMTV-PyMT mice shown in (**d**). Mann–Whitney *U*-test. **f** Representative TMEM Activity-MRI maps of mammary carcinoma tumors by magnetic resonance imaging (MRI) from MMTV-PyMT mice treated with clodronate (second column) or vehicle control (first column). Scale = 1 mm. **g** Quantification of TMEM-mediated vascular opening events (TMEM doorway activity), assessed via TMEM Activity-MRI assay in MMTV-PyMT mice shown in (**f**). Mann–Whitney *U*-test. **h** Correlation between TMEM score (as quantified in **e**) and TMEM Activity-MRI (as quantified in **g**) in MMTV-PyMT mice treated with either control or clodronate liposomes. Spearman’s rank correlation coefficient. **i** Circulating tumor cell (CTC) counts in MMTV-PyMT mice treated with either control or clodronate liposomes. Mann–Whitney *U*-test. **j** Correlation of TMEM activity-MRI score with circulating tumor cells (CTCs) in MMTV-PyMT mice, treated with either control or clodronate liposomes. Spearman’s rank correlation coefficient. Error bars: standard deviation (SD).

TMEM doorways are dynamic structures, and as such, TMEM doorway activity oscillates in time^1^. Multiphoton intravital imaging studies in live animal tumors suggest that a higher density of TMEM doorways in the tumor microenvironment proportionally corresponds to a higher probability of TAVO occurrence^1^. Based on this premise, along with data shown in Fig. 1j–l, we reasoned that targeted suppression of TMEM doorway formation should overall reduce TMEM Activity-MRI, because fewer TMEM doorways would provide fewer TAVO events per voxel. To suppress TMEM doorway formation, we adopted the treatment with clodronate liposomes, a pharmacologic macrophage depletion strategy that reduces TMEM doorways in mice^37^. Clodronate liposomes or vehicle control (i.e., PBS liposomes) were administered for two weeks in 7-week-old PyMT mice with palpable tumors, and, after the completion of treatment, mice were subjected to TMEM Activity-MRI, TMEM score, and circulating tumor cells (CTC) measurements (Fig. 2a). As a positive control, clodronate-treated mice presented with significantly (*p* < 0.01; Mann–Whitney *U*-test) fewer macrophages overall, as assessed by IBA1 immunohistochemistry (Fig. 2b, c and Supplementary Fig. 3b). Because macrophages are integral components of the TMEM doorway cell triads^4,38^, we also confirmed significantly (*p* < 0.05; Mann–Whitney *U*-test) fewer TMEM doorways upon clodronate treatment (Fig. 2d, e), in line with the clodronate-mediated macrophage depletion (Fig. 2b, c). Despite the significant depletion of macrophages in clodronate-treated mice, we did not observe any significant changes in histological features between vehicle- and clodronate-treated animals (Supplementary Fig. 1). Importantly, however, TMEM Activity-MRI was significantly (*p* < 0.05; Mann–Whitney *U*-test) reduced in the clodronate-treated mice (Fig. 2f, g), indicating that the reduction of TMEM doorways via elimination of tumor-associated macrophages may affect the number of TAVO events per voxel. Clodronate treatment slightly affected tumor growth at the endpoint (Supplementary Fig. 4a–c), but this did not bias the measurement of TMEM Activity-MRI, because the variable represents a ratio of hyper-intense voxels to the total number of tumor voxels and thus is unaffected by tumor size. Moreover, TMEM Activity-MRI correlated significantly (*p* < 0.05; Spearman rho = 0.84; Spearman’s rank correlation) with TMEM score (Fig. 2h), suggesting a linear/proportional correlation between TMEM score and TMEM Activity-MRI, as we hypothesized. As mentioned above, an increased number of TMEM scores in the tumor microenvironment is expected to correlate with an increased probability of TAVO events and, as such, cancer cell dissemination. We thus finally measured circulating tumor cells (CTCs) and found that clodronate-mediated macrophage depletion resulted in a significant (*p* < 0.05; Mann–Whitney *U*-test) reduction of CTCs (Fig. 2i). In line with this, TMEM Activity-MRI also correlated significantly (*p* < 0.05; Spearman rho = 0.82; Spearman’s rank correlation) with the number of CTCs (Fig. 2j). The data presented in this section collectively suggest that TMEM Activity-MRI assesses TMEM-dependent metastatic dissemination. In this section, clodronate treatment was not performed in experimental mice for purposes of proposing clinical intervention but only for purposes of indirectly suppressing TMEM doorway assembly and activity and confirming that such suppression could be detected in the newly established TMEM Activity-MRI assay.

### Rebastinib inhibition of TMEM doorway function decreases TMEM Activity-MRI

**Fig. 3:**
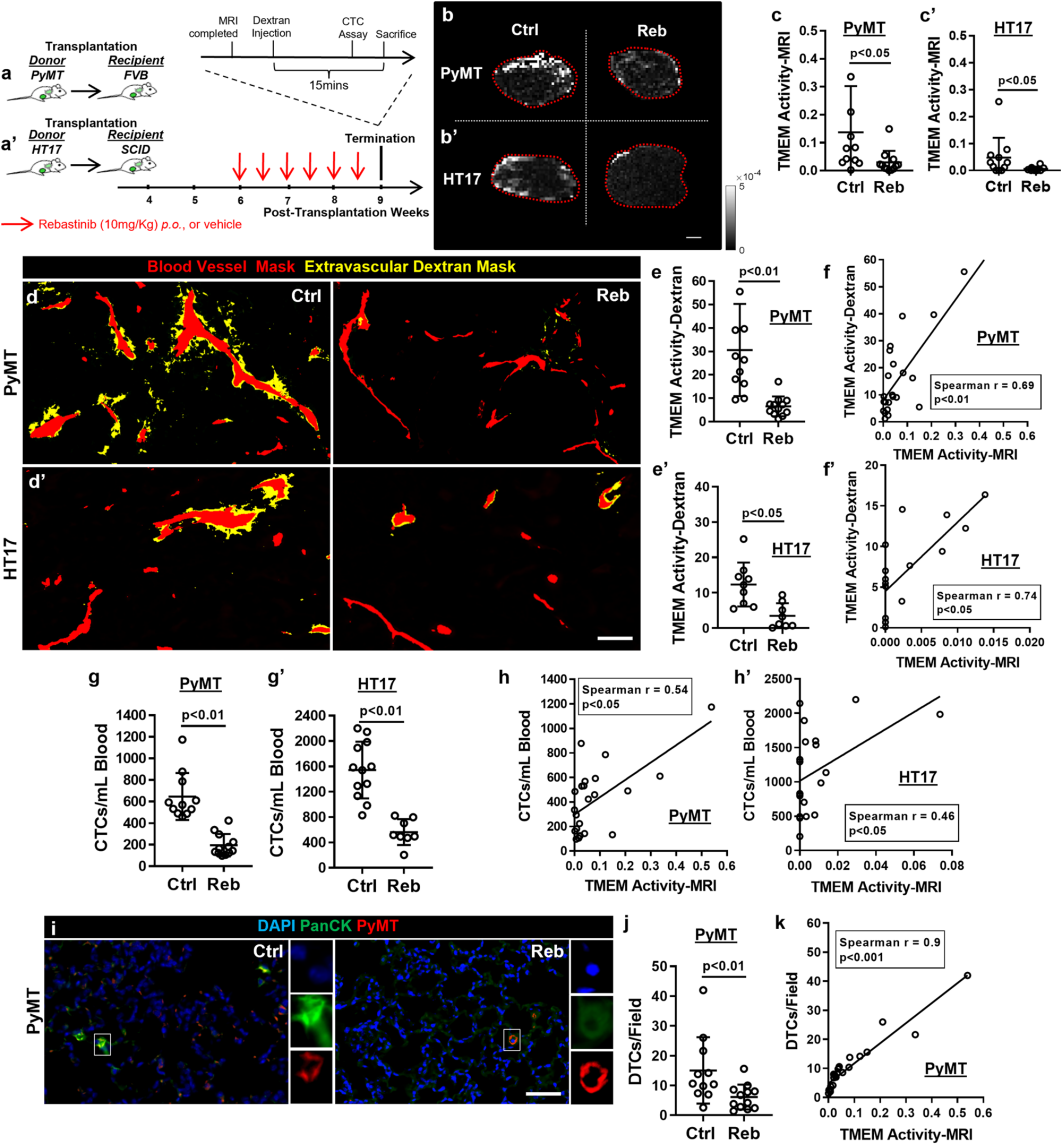
TMEM activity-MRI corresponds to metastatic dissemination endpoints in a mouse model of spontaneous breast carcinoma (MMTV-PyMT; a–k) and a breast cancer patient-derived xenograft (HT17; a′–h′), and is suppressed by the specific TMEM doorway inhibitor, rebastinib. **a–a**′ Experimental strategy and mouse cohort composition for syngeneic (A) and xenogeneic (A′) mouse models of breast carcinoma. PyMT polyoma middle-T antigen, CTC circulating tumor cell, SCID severe combined immunodeficiency, FVB friend virus B. **b–b**′ Representative TMEM Activity-MRI maps of mammary carcinoma tumors by magnetic resonance imaging (MRI) from PyMT (B) and HT17 (B′) mice treated with rebastinib (second column) or vehicle control (first column). Scale = 1 mm. **c–c**′ Quantification of TMEM-mediated vascular opening events (TMEM doorway activity), assessed via TMEM Activity-MRI assay, in PyMT (**c**) and HT17 (**c**′) mice. Mann–Whitney *U*-test. **d–d**′ Binarized images of extravascular dextran in mammary carcinoma tumors from PyMT (**d**) and HT17 (**d**′) mice treated with rebastinib (second column) or vehicle control (first column). Scale = 20 um. **e-e**′ Quantification of TAVO events (a.k.a. TMEM activity), assessed via the TMEM activity-dextran assay, in PyMT (**e**) and HT17 (**e**′) mice. Mann–Whitney *U*-test. **f–f**′ Correlation of TMEM doorway activity scores, as quantified with the TMEM activity-dextran and TMEM activity-MRI assays, in PyMT (**f**) and HT17 (**f**′) mice, treated with either rebastinib or vehicle control. Spearman’s rank correlation coefficient. **g–g**′ Circulating tumor cell (CTC) counts in PyMT (**g**) and HT17 (**g**′) mice treated with either rebastinib or vehicle control. Mann–Whitney *U*-test. **h–h**′ Correlation of TMEM activity-MRI score with circulating tumor cells (CTCs) in PyMT (**h**) and HT17 (**h**′) mice, treated with either rebastinib or vehicle control. Spearman’s rank correlation coefficient. **i** Multichannel immunofluorescence of disseminated tumor cells (DTCs) in MMTV-PyMT mice treated with either rebastinib (right panel) or vehicle control (left panel), as assessed by co-staining of the PyMT antigen, Pancytokeratin (PanCK) and DAPI. Magnified inserts show individual fluorescent channels for the cells outlined with squared boxes in the main images. Scale = 40 um. **j** Quantification of disseminated tumor cells (DTCs) in MMTV-PyMT mice, treated with either rebastinib or vehicle control. Mann– Whitney *U*-test. **k** Correlation of TMEM activity-MRI score with disseminated tumor cells (DTCs) in PyMT mice, treated with either rebastinib or vehicle control. Spearman’s rank correlation coefficient. Error bars: standard deviation (SD).

Prior studies have demonstrated that TMEM doorways induce localized and transient vascular opening associated with tumor cell intravasation, which are both tightly regulated by the TIE2^+^ macrophage at TMEM doorways^1^. Indeed, TMEM doorway activity can be suppressed by targeting the TIE2 signaling pathway in perivascular macrophages^5,6^. The pharmacological suppression of the TIE2 signaling pathway in macrophages at TMEM doorways can be achieved by the specific TIE2 inhibitor, rebastinib, and at doses which demonstrate minimal off-target effects based on in vivo-relevant cellular assays measuring inhibition of kinase activity^5,6^. To examine if TMEM Activity-MRI is indeed indicative of TAVO events, we tested the TMEM Activity-MRI’s ability to detect rebastinib-mediated suppression of TMEM activity. We used two independent mouse models of breast carcinoma, both of which received either rebastinib or vehicle control, to alter TMEM doorway activity. The first model was developed via syngeneic transplantation of PyMT tumors from late-stage PyMT donors into wild-type FVB hosts (Fig. 3a). The second model was developed via xenogeneic transplantation of patient-derived HT17 tumor chunks [previously established from an estrogen receptor-negative (ER-) breast cancer patient^39^] into immunocompromised SCID hosts (Fig. 3a′). Both animal models have been detailed previously in studies involving TMEM-dependent cancer cell dissemination and metastasis^1,5,36,39^. Following the development of palpable tumors in both models (4–6 weeks after transplantation), mice received a 3-week treatment with rebastinib or vehicle [administration protocol detailed in ref. ^5^], followed by an MRI session and subsequent measurement of TMEM Activity-MRI and other metastatic endpoints (Fig. 3a, a′). Histological assessment of the resected PyMT and HT17 tumors, as expected, revealed features of late-stage carcinomas (Supplementary Fig. 1), consistent with prior observations^5^. Importantly, TMEM Activity-MRI was significantly (*p* < 0.05; Mann–Whitney *U*-test) reduced in both the PyMT (Fig. 3b, c) and the HT17 (Fig. 3b, c) rebastinib-treated mice compared to vehicle-treated mice. It should be underscored that rebastinib, as opposed to the clodronate treatment experiments described above (Fig. 2), specifically affects the function, but not the assembly of TMEM doorways in the breast tumor microenvironment^5,6^. Indeed, the TMEM doorway score remained unaltered between vehicle- and rebastinib-treated animals for both the PyMT and the HT17 models (Supplementary Fig. 5a, b). Interestingly, in the absence of an extrinsic factor (e.g., rebastinib) capable of modulating TMEM doorway activity (as, for example, in the transition from early to late breast carcinoma where TMEM doorway activity increases with TMEM doorway score), TMEM activity-MRI correlates well with TMEM doorway score (Fig. 1l). However, upon treatment of breast carcinomas with rebastinib, we did not observe any correlation between TMEM score and TMEM activity-MRI in either of the two models tested (Supplementary Fig. 5c). This observation was completely expected since rebastinib is known to inhibit TMEM doorway activity without affecting the assembly of new, or the breakdown of existing, TMEM doorways^5^. As a consequence, these observations collectively show that TMEM Activity-MRI detects the significant reduction of TMEM doorway activity in rebastinib-treated mice. This observation further indicates that TMEM Activity-MRI better mirrors the activity, and not as much the assembly, of TMEM doorways in the tumor microenvironment.

### TMEM Activity-MRI correlates with established endpoints of TMEM doorway-associated vascular opening and metastatic dissemination

Previously, we have developed a multichannel immunofluorescence assay to specifically visualize and quantify localized TAVO events in breast cancer^40^. This assay, here termed “TMEM Activity-Dextran,” is based on the intravenous injection of high-molecular-weight (155 kDa) dextran conjugated to tetramethylrhodamine (TMR) in experimental mice a few minutes before the termination of the experiment. In intact blood vessels, the fluorescent probe is restrained in the vascular lumens, because its molecular weight prevents it from passing between the endothelial cells^1^. However, under circumstances where endothelial cell tight junctions are dissolved as a result of TMEM doorway activity^1^, the fluorescent probe can leak into the tumor tissue and be visualized and quantified as a high TMR signal accumulation in the perivascular space^40^. Here, we adapted the TMEM Activity-Dextran assay by (following the termination of the MRI session) injecting Dextran-TMR directly into the tail vein and sacrificing the mice 15’ later (Fig. 3a-a′ and Supplementary Fig. 6a). It should be noted that the appearance of vascular profiles, as well as the baseline values of the TMEM Activity-Dextran assay in PyMT mice following an MRI session (adapted protocol with the addition of GBCA), are similar to those of PyMT mice not subjected to an MRI session [published protocol without the addition of GBCA as in ref. ^40^] (Supplementary fig. 6a, b). Moreover, no extravascular dextran staining is detected in tissue sections of mice receiving GBCA without the dextran injection (Supplementary Figs. 6c), indicating the presence of GBCA in the peripheral circulation of the experimental mice does not interfere with the endpoint measurement of the TMEM Activity-Dextran assay. Importantly, we noticed that TMEM Activity-Dextran was significantly (*p* < 0.05; Mann–Whitney *U*-test) suppressed in rebastinib-treated compared to vehicle-treated PyMT (Fig. 3d, e) and HT17 (Fig. 3d′, e′) mice. In support of this observation, TMEM Activity-MRI significantly (*p* < 0.05; Spearman’s rank correlation) correlated with TMEM Activity-Dextran in both PyMT (Spearman rho = 0.69) and HT17 (Spearman rho = 0.74) models (Fig. 3f, f′), further strengthening the notion that TMEM Activity-MRI captures TMEM doorway activity within the tumor microenvironment.

TMEM doorway function results not only in the localized and transient vascular opening but also in the intravasation of highly invasive, highly migratory tumor cells into the peripheral circulation^1^. Consistent with these observations, the administration of rebastinib in both PyMT (Fig. 3g) and HT17 (Fig. 3g′) mice significantly (*p* < 0.01; Mann–Whitney *U*-test) decreased the number of CTCs in the peripheral circulation. Importantly, TMEM Activity-MRI significantly (*p* < 0.05; Spearman’s rank correlation) correlated with CTCs in both the PyMT (Spearman rho = 0.54) and the HT17 (Spearman rho = 0.46) models (Fig. 3h, h′). The correlation of TMEM Activity-Dextran with TMEM Activity-MRI is stronger than that of CTCs with TMEM Activity-MRI (compare Fig. 3f, f′ with Fig. 3h, h′). This is in consistent with the fact that the number of CTCs in the peripheral circulation does not only depend on TAVO events but may also be determined by other factors, such as the dwell time of CTCs in the circulation resulting from prolonged survival and evasion of CTCs from innate/adaptive immunity^41,42^.

Finally, we assessed the correlation of the newly described TME Activity-MRI measurement with the most direct metastatic dissemination outcome, the presence of disseminated tumor cells (DTCs) in secondary sites, in particular the lungs. DTCs were detected as single cells in the lung parenchyma, co-expressing pancytokeratin (PanCK^+^), which is a generic epithelial marker, and the PyMT antigen (PyMT^+^), which is specific to cancer cells in the MMTV-PyMT model (Fig. 3i). As expected, rebastinib treatment results in significantly (*p* < 0.01; Mann–Whitney *U*-test) fewer DTCs in the lung parenchyma of PyMT mice (Fig. 3j). In support to this finding, TMEM Activity-MRI significantly correlated (*p* < 0.01; Spearman rho = 0.9; Spearman’s rank correlation) with DTCs in this model (Fig. 3k). Taken together, the data presented in this section show that TMEM Activity-MRI is an accurate measure of TMEM doorway function during dissemination and indicative of the prometastatic potential of breast carcinomas.

### TMEM activity-MRI reveals that mammalian-enabled (MENA) is not essential for TMEM doorway-associated vascular opening but is necessary for cancer cell dissemination

So far, we demonstrated that TMEM Activity-MRI is a potentially useful MRI-based measurement of TMEM doorway activity by using diverse mouse models of breast carcinoma with perturbations in either the TMEM doorway number (Fig. 2) or TMEM doorway function/activity (Fig. 3). Next, we sought to investigate whether this novel TMEM Activity-MRI assay could be used in mechanistic studies related to the molecular basis of cancer cell dissemination.

**Fig. 4:**
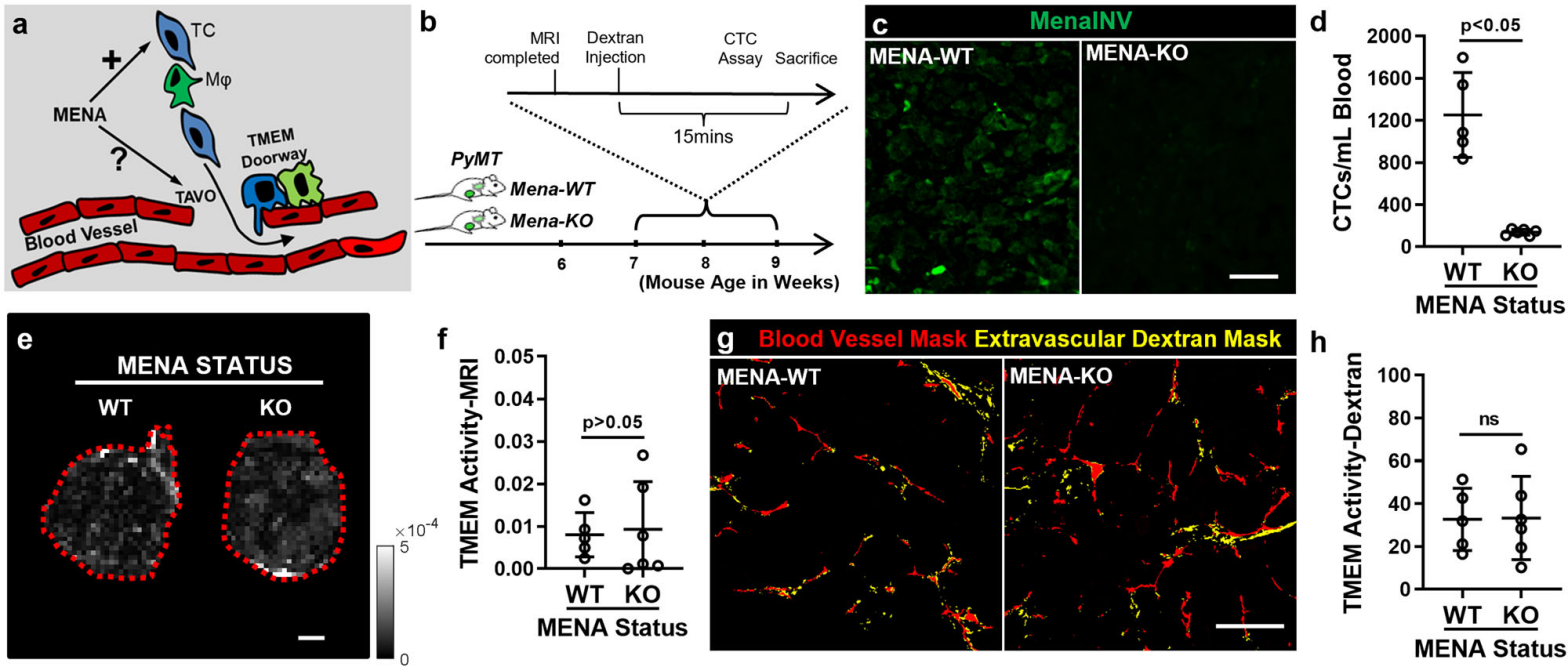
TMEM activity-MRI demonstrates that MENA is not necessary for TMEM-associated vascular opening events but is essential for cancer cell dissemination. **a** Experimental hypothesis under investigation using the TMEM Activity-MRI assay. The illustration displays two prerequisites of metastatic dissemination: TMEM doorway [(a cell triad composed of a macrophage (green), a tumor cell (blue), and an endothelial cell (red)], and the invasive/migratory tumor cell subset migrating alongside macrophages towards underlying TMEM doorways. Although it is known from prior literature (see text for details) that MENA is necessary for inducing invasive and migratory behavior to the prometastatic tumor cells, it is not known if MENA is also necessary for TMEM-associated vascular opening (TAVO), as depicted with the question mark. **b** Experimental strategy and mouse cohort composition for MMTV-PyMT (MENA^+/+^ and MENA^−/−^) mice. PyMT polyoma middle-T antigen, CTC circulating tumor cell. **c** Representative images of MENA^INV^ immunofluorescence from MENA^+/+^ (MENA-WT) and MENA^−/−^ (MENA-KO) MMTV-PyMT mice. Scale = 50 um. **d** Circulating tumor cell (CTC) counts in MENA^+/+^ (MENA-WT) and MENA^−/−^ (MENA-KO) MMTV-PyMT mice. Mann–Whitney *U*-test. **e** Representative TMEM Activity-MRI maps of mammary carcinoma tumors by magnetic resonance imaging (MRI) from MENA^+/+^ (MENA-WT) (first column) and MENA^−/−^ (MENA-KO) (second column) MMTV-PyMT mice. Scale = 1 mm. **f** Quantification of TMEM-mediated vascular opening events (TMEM doorway activity), as assessed via the TMEM Activity-MRI assay, in MENA^+/+^ (MENA-WT) and MENA^−/−^ (MENA-KO) MMTV-PyMT mice. Mann–Whitney *U*-test. **g** Binarized (thresholded) images of extravascular dextran in mammary carcinoma tumors from MENA^+/+^ (MENA-WT) and MENA^−/−^ (MENA-KO) MMTV-PyMT mice. Scale = 200 um. **h** Quantification of TAVO events (a.k.a. TMEM activity), as assessed via the TMEM Activity-dextran assay, in MENA^+/+^ (MENA-WT) and MENA^−/−^ (MENA-KO) MMTV-PyMT mice. Mann–Whitney *U*-test. Error bars: standard deviation (SD).

Amid the two main prerequisites of cancer cell dissemination, i.e., the presence of TMEM doorway and the presence of a proinvasive/promigratory cancer cell subpopulation, prior data have shown that the invasive isoform of the actin-regulatory protein MENA, MENA^INV^, is critical for the latter prerequisite^4,38^. Indeed, PyMT mice lacking the MENA gene (MENA-KO) fail to establish metastatic disease^43^. Moreover, MENA^INV^ is necessary for the transendothelial migration of tumor cells since it regulates the development and maturation of invadopodia, which are essential cytoplasmic protrusions for the migratory/invasive process^44–47^. Nevertheless, it is not clear whether MENA simply defines the invasive properties of the prometastatic cancer cell subpopulation or whether it is also necessary for TMEM doorway activity (Fig. 4a). The newly developed TMEM Activity-MRI assay is ideal to answer this question, as TMEM Activity-MRI is specific for measuring TAVO events and TMEM doorway activity. In this regard, we crossed MMTV-PyMT mice with MENA heterozygotes to develop MMTV-PyMT MENA^−/−^ (herewith referred to as MENA-KO mice), while the MMTV-PyMT MENA^+/+^ (MENA-WT) litter served as the wild-type control (Fig. 4b and Supplementary Fig. 1). We subjected 7–9-week-old MENA-KO and MENA-WT mice bearing an average diameter of PyMT tumors of ~0.5 cm, to TMEM Activity-MRI assay and experimental endpoints of metastatic dissemination (Fig. 4b). Retrospectively, we confirmed that the resected MENA-KO tumors did not express the prometastatic MENA^INV^ isoform using immunofluorescence (Fig. 4c), and, as expected from our prior work^43^, displayed significant (*p* < 0.05; Mann–Whitney *U*-test) suppression of CTCs, compared to MENA-WT mice (Fig. 4d).

Surprisingly, we did not observe any difference (*p* > 0.05; Mann–Whitney *U*-test) in TMEM Activity-MRI between MENA-WT and MENA-KO mice (Fig. 4e, f), implying that MENA is not directly involved in the regulation of TMEM doorway activity, but may exclusively contribute to the establishment of the proinvasive/promigratory cancer cell subpopulation that disseminates via TMEM doorways^46–49^. To confirm that the genetic elimination of the MENA gene in MMTV-PyMT mice did not interfere with the MRI assay giving false-negative results, we also evaluated these observations by using TMEM Activity-Dextran in the same animals that were imaged with MRI (Fig. 4g, h). Similarly, there was no significant (*p* > 0.05; Mann–Whitney *U*-test) difference in TMEM Activity-Dextran between MENA-WT and MENA-KO mice (Fig. 4g, h), suggesting that TMEM Activity-MRI is indeed specific for measuring TAVO events. Overall, these data served a dual purpose. Foremost, they indicate that MENA is not necessary for TMEM doorway-dependent vascular opening, despite it being necessary for metastatic dissemination (Fig. 4a). Second, these data provide an accurate proof of principle that TMEM Activity-MRI could be generally used to investigate the molecular mechanisms behind cancer cell intravasation and endothelial permeability during metastasis.

### Translational relevance of TMEM activity-MRI: Potential utility as a companion diagnostic

**Fig. 5:**
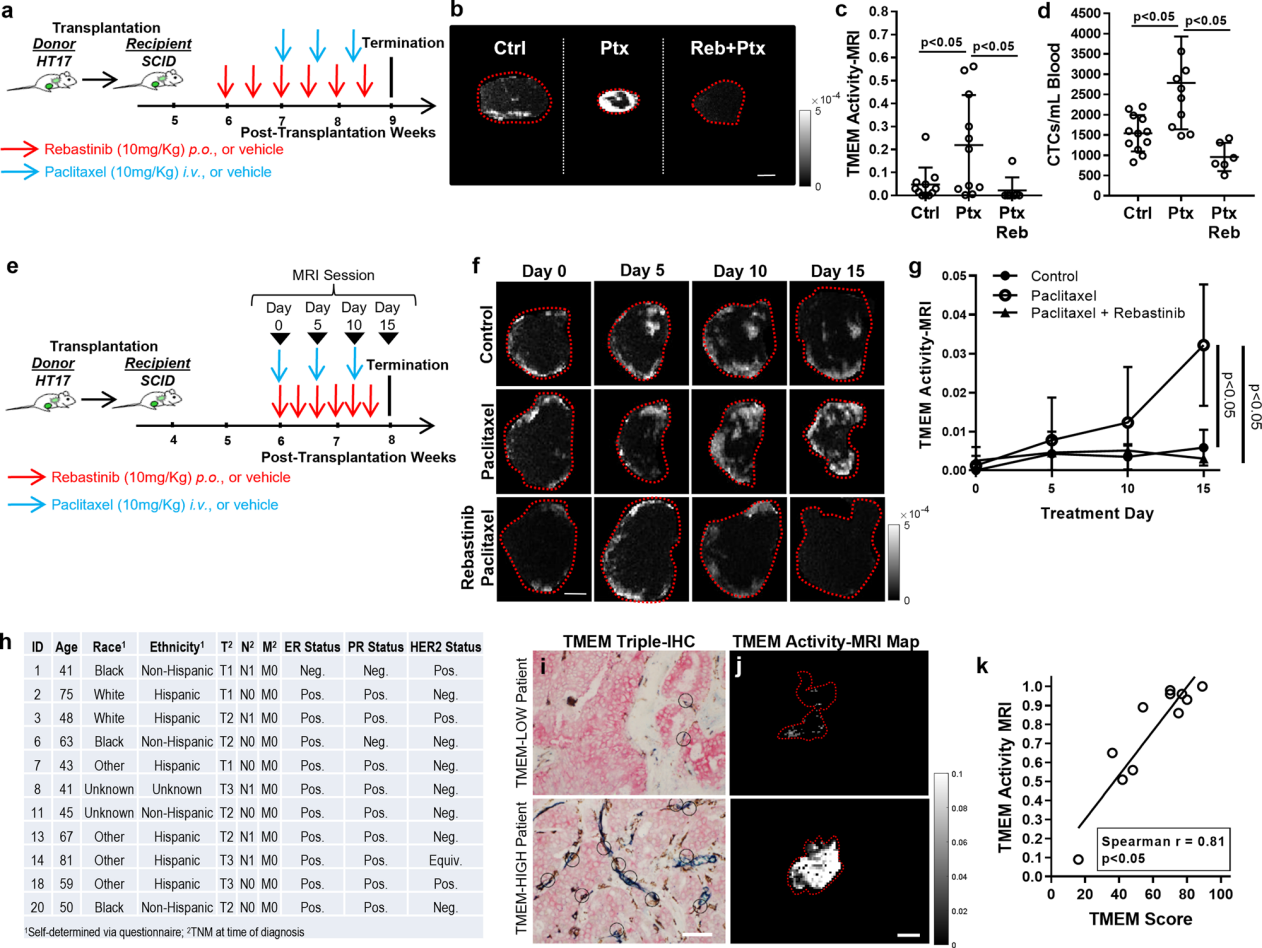
Translational significance of the newly developed TMEM activity-MRI assay. **a** Experimental strategy and mouse cohort composition for HT17 breast cancer patient-derived xenograft. SCID severe combined immunodeficiency. **b** Representative TMEM activity-MRI maps of mammary carcinoma tumors by magnetic resonance imaging (MRI) in HT17 mice treated with vehicle control (Ctrl; left panel), paclitaxel chemotherapy (Ptx; middle panel), or paclitaxel plus rebastinib (Reb + Ptx; right panel). Scale = 1 mm. **c** Quantification of TAVO events (a.k.a. TMEM activity), assessed via the TMEM activity-MRI assay, in HT17 mice shown in (**b**). Kruskal–Wallis analysis of variance with Mann–Whitney *U*-test for post hoc analysis. **d** Circulating tumor cells (CTCs) in HT17 Mice treated with chemotherapy alone or chemotherapy in combination with rebastinib. Kruskal–Wallis analysis of variance with Mann–Whitney *U*-test for post hoc analysis. **e** Experimental strategy and mouse cohort composition for HT17 breast cancer patient-derived xenograft. SCID, severe combined immunodeficiency. Black arrowheads indicate time-points, in which TMEM activity-MRI was assessed. **f** Representative TMEM activity-MRI maps of mammary carcinoma tumors by magnetic resonance imaging (MRI) in HT17 mice treated with vehicle control (first row), paclitaxel chemotherapy (second row), or paclitaxel plus rebastinib (third row) across multiple time-points (first column, day 0; second column, day 5; third column, day 10; fourth column, day 15). Scale = 1 mm. **g** Quantification of TMEM-mediated vascular leakiness (TMEM activity), assessed via the TMEM activity-MRI assay across multiple time-points in the HT17 mice shown in (**f**). **h** Breast cancer patient demographic and histopathologic data. **i**, **j** Representative images from TMEM triple-stain immunofluorescence (**i**) and TMEM Activity-MRI maps of the corresponding tumor ROIs (**j**) in the pilot patient cohort of breast carcinoma, here presented as “low” TMEM doorway score patient (first row), and “high” TMEM doorway score patient (second row). Scale = 100 um (**i**) and 1 cm (**j**). **k** Correlation of TMEM activity-MRI score with TMEM doorway score in the pilot patient cohort. Spearman’s rank correlation coefficient. Error bars: standard deviation (SD).

Finally, we examined if TMEM activity-MRI could be extended into the preclinical setting to provide any insights into its potential clinical importance. Our group has previously demonstrated that treatment with neoadjuvant paclitaxel or doxorubicin/cyclophosphamide can significantly increase TMEM doorway assembly and activity as a result of the infiltration of prometastatic macrophages in both mouse and human breast cancer^5^. Such modifications in the tumor microenvironment are capable of delaying tumor growth in the short term but otherwise obfuscate the long-term clinical benefits of chemotherapy treatment. These may also contribute to the observed distant relapse following treatment with chemotherapy in some patients^50–52^. Because not all breast cancer patients respond with the development of the aforementioned prometastatic macrophage infiltration upon treatment with chemotherapy, we have previously indicated the importance of developing non-invasive approaches for monitoring the tumor microenvironment while patients undergo pre-operative chemotherapy^50–52^. We reasoned that TMEM activity-MRI is a promising tool for this purpose because it correlates with TMEM doorway function in all preclinical models tested thus far (Figs. 1–3), and importantly, TMEM Activity-MRI can be acquired in a non-invasive manner. To examine if TMEM Activity-MRI can capture chemotherapy-mediated changes in TMEM doorway function in breast tumors, we again utilized the HT17 patient-derived xenograft (PDX) model, which demonstrates excellent preclinical utility (Supplementary Fig. 1), especially in the context of chemotherapy-induced metastasis^5^. It should be noted that the HT17 mice treated with paclitaxel, with or without rebastinib, were generated from the same cohort as the HT17 mice shown in Fig. 2; as a result, the same untreated animal group could be re-graphed as a reference group to demonstrate baseline TMEM Activity-MRI values (Fig. 5a). Importantly, our results indicate that TMEM activity-MRI is significantly increased (*p* < 0.05; Kruskal–Wallis analysis of variance; post hoc analysis: Mann–Whitney *U*-test) in HT17 mice receiving neoadjuvant paclitaxel when compared to the vehicle controls (Fig. 5b, c). As expected, the addition of rebastinib in paclitaxel-treated HT17 mice results in a significant (*p* < 0.05; Kruskal–Wallis analysis of variance; post hoc analysis: Mann–Whitney *U*-test) reduction of TMEM Activity-MRI (Fig. 5b) and CTCs (Fig. 5d).

Based on these preliminary data, we reasoned that TMEM Activity-MRI could be utilized as a companion diagnostic to monitor the effects of chemotherapy in breast cancer patients. To evaluate such potential in a preclinical setting, we designed another mouse carcinoma study, again using the HT17 patient-derived xenograft (Supplementary Fig. 1). In a longitudinal fashion, HT17 mice either received paclitaxel chemotherapy alone or paclitaxel along with rebastinib, while TMEM Activity-MRI was measured in frequent intervals to mimic the clinical scenario of monitoring breast cancer patients in the course of neoadjuvant treatment (Fig. 5e). As expected^5^, we found that paclitaxel chemotherapy significantly (*p* < 0.05; repeated-measures ANOVA) increased TMEM Activity-MRI during the course of treatment, compared to vehicle alone (Fig. 5f, g). Importantly, however, the co-administration of rebastinib with paclitaxel prevented the expected chemotherapy-induced increase of TMEM Activity-MRI, thus bringing the raw TMEM Activity-MRI values down to the same levels as the mice treated with vehicle controls (Fig. 5f, g). These observations suggest that the newly established TMEM Activity-MRI measurement represents a potential surrogate of TMEM doorway activity in preclinical animal models of breast carcinoma, thus demonstrating the significant potential for clinical utility.

TMEM doorway activity assays are not currently available in human clinical practice because it is not possible to inject fluorescent dextran in human patients to evaluate TMEM Activity-Dextran in a clinical setting. Thus, we evaluated the possibility that the new TMEM Activity-MRI measurement could serve as a non-invasive surrogate for TMEM doorway activity and, therefore, cancer cell dissemination in humans. Thus, we assembled a “pilot” patient cohort that accrued 11 patients without distant metastasis who had a wide distribution in tumor size (T1 = 3, T2 = 5, T1 = 3) and axillary lymph node involvement (node-positive = 5). This cohort included patients with a wide range of TMEM doorway scores determined by using the standardized TMEM scoring method on tissue sections (Fig. 5h), expected to present disparate metastatic risk according to prior clinical investigations^2,3,7^. Tumor ROI acquisition and TMEM Activity-MRI calculations were performed in an analogous fashion to the mouse protocol, albeit with minor modifications, as described in Materials and Methods. As representative examples, TMEM doorway immunostaining images (with either low or high TMEM scores) from biopsies obtained from the tumor site prior to the MRI session are presented along with corresponding MRI analyses (Fig. 5i, j). In this pilot patient cohort, TMEM Activity-MRI correlated positively and significantly with TMEM score (*p* < 0.05; Spearman rho = 0.81; Spearman’s Rank Correlation), irrespective of tumor size, age, race, ethnicity, or lymph node status (Fig. 5k). Along with the animal preclinical data shown in this study, this human pilot study additionally indicates that TMEM Activity-MRI could serve as a companion diagnostic in the clinical management of breast cancer patients.

## Discussion

It has long been known that TMEM doorway activity correlates with increased metastatic potential in preclinical mouse models of breast carcinoma^4,38,53^. Clinical investigations have since demonstrated that an increased number of TMEM doorways correlates with increased metastatic risk in breast cancer patients^2,3,7^. However, protocols for measuring TMEM doorway activity, and therefore metastatic risk, in a non-invasive manner (i.e., without the surgical extraction of the primary tumor or a core biopsy) do not exist in the clinical setting. These observations have together inspired the pursuit of a novel, non-invasive tool/assay for the successful measurement of TMEM doorway activity in breast cancer patients, which could theoretically be embedded in standard-of-care clinical practice. In this regard, this study focused on the algorithmic development and validation of the *TMEM Activity-MRI* assay, a dynamic contrast-enhanced first-pass deconvolution MRI approach that measures TAVO events, known to biologically correlate with cancer cell intravasation and dissemination in the peripheral circulation. Furthermore, this study provides critical insights for the subsequent transfer of the TMEM Activity-MRI assay to the clinical setting, with the vision of facilitating treatment decision-making for breast cancer patients undergoing neoadjuvant treatment.

While the translational potential of the TMEM Activity-MRI assay is highlighted in the current research-oriented pilot study, the clinical potential of the assay requires its incorporation into the standard-of-care MRI exam. Indeed, the proposed TMEM Activity-MRI assay is highly compatible with standard-of-care MRI procedures, requiring the collection of dynamic (high temporal and spatial) resolution images starting at contrast infusion and lasting for 90s of contrast circulation. Traditionally, standard-of-care images are acquired after this time period. In total, this acquisition and the accompanying pre-contrast T1-relaxation quantification adds roughly 3 to-4 min to the entire clinical exam, does not alter the clinical effectiveness, and therefore can be routinely added to the stand-of-care exam. MRI sequences necessary for this rapid acquisition of dynamic data are routinely available on most clinical high-field (1.5 and 3 Tesla) MRI systems, so these methods have been easily incorporated into clinical practice in the clinical setting. In conclusion, a single combined MRI exam, which includes both the standard-of-care clinical assessment of the patient and our proposed TMEM Activity-MRI assay, will not delay the patient’s clinical assessment or induce additional discomfort to the patient and could be readily available for use in the clinic as a companion diagnostic.

Several studies have previously reported on the use of dynamic contrast-enhanced (DCE) MRI to estimate the metastatic state and outcome of breast tumors^54–56^. Typically, these studies have used the volume transfer constant between the blood plasma and the extravascular extracellular space (K_trans_) as a marker, which evaluates the full wash-in of the contrast agent^57,58^. In contrast, TMEM Activity-MRI only uses the extravasation of GBCA during the first pass through the vasculature, exclusively corresponding to the tightly controlled vascular opening of the TMEM doorway. As such, TMEM doorway-independent mechanisms of vascular leakage (i.e., necrosis) are efficiently isolated in our established MRI measurement, as shown in Fig. 1c (yellow arrow). Therefore, compared to prior methods^54–56^, TMEM activity-MRI is more efficient in eliminating background signals from various sources not associated with the active process of cancer cell intravasation and dissemination.

TMEM doorways are composed of three individual cells in direct and stable physical contact: a perivascular macrophage, an endothelial cell, and a tumor cell highly expressing the actin-regulatory protein Mena^2,4,53^. As such, a single voxel in a TMEM Activity-MRI map can contain multiple TMEM doorways which are approximately 40–60 um in diameter (the approximate average diameter of a single TMEM doorway), and the observed signal intensity is likely the result of cumulative signals from multiple active TMEM doorways in the tumor microenvironment. Despite the overall lower resolution of MRI, the hyper-intense voxels within a TMEM Activity-MRI map likely correspond to large densities (i.e., hotspots) of active TMEM doorways, given that background signal from other sources is very low, as explained above. However, super-resolution kinetic analysis of TMEM doorway activity via multiphoton intravital imaging has previously demonstrated that TMEM doorways remain open for only ~20 min before the endothelium is spontaneously re-sealed^1^. It is thus clear that the hyper-intense voxels within the TMEM Activity-MRI map can capture regions where metastatic dissemination is currently active. This measurement, therefore, offers exciting possibilities for physician-based monitoring of the metastatic potential in the era of personalized medicine.

Metastasis is the primary cause of death in breast cancer, yet no clinically validated imaging modalities are available that reflect the ability of primary breast cancer to metastasize. Although screening mammography has contributed to greater detection rates and reduced breast cancer mortality, it also results in over-diagnosis or detection of cancers that pose no threat to life. There is, therefore, an unmet need to develop screening modalities, used as a primary screening test, or a reflex test after initial screening mammography, that may distinguish non-lethal versus potentially lethal cancers. Multiparameter gene expression assays, including Oncotype DX Recurrence Score^59^, MammaPrint^®TM^, Prosigna™, and Breast Cancer Index℠)^60^ provide similar prognostic information that is driven largely by proliferation and estrogen-dependent genes and not by the intrinsic propensity of tumor cells to metastasize or interact with their microenvironment^61–63^. Interestingly, TMEM doorway Score correlates poorly with the Oncotype DX Recurrence Score, captures different biologic information, and provides complementary prognostic information^7^. Thus, the TMEM doorway score, and its highly correlated TMEM-MRI score, offer the potential to more accurately determine prognosis regarding recurrence beyond what is possible for lower ranges of Oncotype DX and other multiparameter gene expression scores. This could lead to treatment decisions that differ from those made based on Multiparameter gene expression assays alone.

Another potential clinical application of TMEM Activity-MRI is as a companion diagnostic to monitor the pharmacodynamic effects of standard cytotoxic therapy and also therapeutic interventions designed to induce a blockade of TMEM doorways. It is known that cytotoxic chemotherapy has profound effects on the tumor microenvironment^50,52,64^, including promoting an influx of proangiogenic M2 macrophages^64–69^ and the formation of TMEM doorways^5,70^. Chemotherapy may also increase the density of cancer cells with high dissemination potential^5,71–73^. Therefore, quantifying the effects of chemotherapy on the dissemination potential of the tumor microenvironment could be used as a pharmacodynamic biomarker to stratify distant metastatic risk in patients with residual disease after neoadjuvant chemotherapy or adding agents such as the Tie2 kinase inhibitor rebastinib that blocks TMEM doorway-mediated cancer cell intravasation. Additional studies are required to further evaluate the clinical validity of TMEM Activity-MRI for distant recurrence risk and its potential clinical utility in breast cancer management.

All the validation experiments performed in this study have consistently confirmed that TMEM Activity-MRI significantly correlates with endpoints of metastatic dissemination, including TMEM doorway activity, circulating tumor cells (CTCs), and disseminated tumor cells (DTCs), suggesting that TMEM Activity-MRI can serve as a specific tool to study the biology of cancer cell dissemination, which is often seen as the rate-limiting step of the metastatic cascade^4,38^. Here, as a proof-of-concept, we documented that MENA expression is not critical for the function of TMEM doorways, even though it is necessary for the generation of a proinvasive/promigratory tumor cell subset (Fig. 4). These observations foster a subsequent question pertaining to the precise MENA-independent mechanism, via which cytoskeletal dynamics at TMEM doorways may regulate endothelial cell opening to facilitate transendothelial migration. Indeed, other members of the Ena/VASP family could play a key role in this process^74,75^ and should be explored in the future to fully appreciate the complex regulation of TAVO events. Interestingly, TMEM doorways are microenvironmental niches that may cultivate induction and maintenance of stemness and localized immunosuppression^76,77^, indicating they may confer suitable niches for the development of immune-privileged, metastatic stem cells^78^. The particularly high correlation observed between TMEM Activity-MRI and DTCs (Fig. 3i–k) is supportive of this notion and further indicates that TMEM Activity-MRI may harbor additional potential in studying the immune tumor microenvironment.

In this study, TMEM Activity-MRI was only measured in primary tumors of mice and humans. However, more recent observations suggest that TMEM doorways do not only assemble in the primary tumor microenvironment but also in local (i.e., lymph nodes) or distant (i.e., lungs) metastatic sites^79–81^. These observations raise the intriguing possibility that metastatic breast tumors may potentially utilize hematogenous routes, based on the assembly of TMEM doorways, to efficiently re-disseminate to tertiary sites after achieving metastatic colonization^80,82,83^. As such, future efforts should establish whether the TMEM Activity-MRI assay presents with any significant translational application in metastatic cancers, as well.

While our study has primarily focused on breast cancer, it is now known that other types of cancer, such as pancreatic neuroendocrine tumors, also utilize TMEM doorways as the primary cancer cell dissemination machinery for the initial steps of the metastatic cascade^6^. Previously, pancreatic tissue and tumors have been successfully visualized using low or high-resolution imaging modalities, including magnetic resonance imaging and multiphoton intravital microscopy^84–88^. It would therefore be extremely interesting to extend our studies into other types of cancer and investigate whether TMEM activity-MRI or an equivalent MRI-based measurement could be universally used as an assessment tool for metastatic potential.

In conclusion, this article describes the development and validation of a novel MRI measurement that correlates with metastatic dissemination in preclinical models of breast carcinoma and breast cancer patients. The technological and conceptual innovation of this newly proposed measurement, herewith known as TMEM Activity-MRI, is the quantification of only the first pass of a gadolinium-based contrast agent (GBCA) into the tumor tissue. This procedure notably eliminates background signals from other sources and highly correlates with the biological activity of cancer cell dissemination doorways, known as TMEM doorways, whose activity is the rate-limiting step of metastatic dissemination. The TMEM Activity-MRI assay is sensitive to various anti-cancer and anti-metastatic therapies (i.e., chemotherapy, TMEM doorway inhibitors, etc.) and has been successfully utilized here to classify patients into high and low-risk individuals for developing metastasis as a non-invasive TMEM score surrogate. We propose that TMEM Activity-MRI could be utilized as a promising companion diagnostic to facilitate physicians with decision-making, especially during treatment, as well as with the clinical management of breast cancer patients.

## Methods

### Animal subjects

#### Ethics statement

All studies involving mice were carried out in accordance with the National Institutes of Health (NIH) regulation concerning the care and use of experimental animals and with the approval of the Institutional Animal Care and Use Committee (IACUC) of Molecular Imaging, Inc. (Ann Arbor, MI), a facility accredited by the Association for Assessment and Accreditation of Laboratory Animal Care (AAALAC), or with the approval of the Albert Einstein College of Medicine Animal Care and Use Committee.

#### MMTV-PyMT (spontaneous model)

Transgenic mice expressing the Polyoma Virus Middle-T (PyMT) antigen under the control of mammary tumor virus long terminal repeat (MMTV-LTR)^31^ were bred in-house at the Albert Einstein College of Medicine (Condeelis lab), maintained on the FVB background, and the resulting tumors could be palpated at ~6 weeks of age. Depending on the experimental question, MMTV-PyMT mice were used in different age groups.

#### MMTV-PyMT (transplantation model)

Syngeneic transplantation models were generated through orthotopic transplantation of 1 mm^3^ tumor chunks from 12–16-week-old MMTV-PyMT donor mice bearing late-stage carcinomas of ~1 cm in diameter into 5–6-week-old FVB recipients. These tumors were not passaged in culture or dissociated but propagated as tumor chunks in vivo^39,43^. The tumor chunk was implanted on the fourth mammary pad on the right side of the recipient mouse.

#### MMTV-PyMT/ Mena^−/−^ (spontaneous model)

Generation of the MMTV-PyMT Mena^−/−^ mice was achieved by crossing MMTV-PyMT mice with MENA heterozygotes as described^43^. The forward and reverse primer sequences used to identify the transmission of the disrupted MENA allele in MENA^−/−^ mice are the following: LACZ-F: CGATCGTAATCACCCGAGTGT; LACZ-R: CCGTGGCCTGACTCATTCC; Enah-3-F: ACCGCAGTCTCCCTTACATAACTTA; Enah-3-R: GCACTGCACTTTTAATCAGGTGTCT.

#### Patient-derived xenograft (PDX) model

The generation of the estrogen receptor-negative (ER^-^) HT17 patient-derived xenograft has been developed in-house, as described^39^. Similar to the PyMT-transplantation model described above, the HT17 tumors were also never passaged in culture or dissociated but only propagated as tumor chunks in vivo *(*passage number <5*)*. Each tumor chunk was implanted on the fourth mammary pad on the right side of severe combined immunodeficiency (SCID) recipient mice. The resulting tumors can be palpated 4–6 weeks following the transplantation.

### Chemicals and reagents administered to mice

#### Rebastinib reconstitution and administration

Rebastinib was reconstituted at a concentration of 10 mg/mL in 0.4% hydroxypropyl methylcellulose (HPMC). Each mouse in the experimental group received p.o. doses of 10 mg/kg rebastinib (100 μL total volume) twice per week, for 4 weeks. The control group received p.o. 100 μL of HPMC.

#### Chemotherapy reconstitution and administration

Mice were treated with the taxane-based chemotherapeutic paclitaxel (Sigma-Aldrich). Paclitaxel was reconstituted at a concentration of 10 mg/mL in 1:1 EtOH:Cremophor-EL (Millipore). Each mouse in the experimental group received an i.v. dose of 10 mg/Kg paclitaxel (200 μL total volume) every 5 days, for a total of three doses. The control group received an i.v. injection of 200 μL 1:1 EtOH:Cremophor-EL.

#### Clodronate and PBS liposome reconstitution and administration

The clodronate liposomes were administered in experimental mice with an i.v. injection at a dose of 5 mL/Kg (200 μL total volume) every other day for a total of seven doses. The control group received an equivalent i.v. injection of PBS liposomes.

#### High-molecular weight (155 kDa) dextran reconstitution and administration

155-kDa Tetramethylrhodamine-Dextran (TMR-Dextran) solution was reconstituted at a concentration of 20 mg/mL in sterile phosphate-buffered saline (PBS). Each mouse in control or experimental groups received 100 μL total volume of TMR-Dextran via right-sided retro-orbital injection, 1-h before sacrifice.

#### Gadolinium reconstitution and administration

The gadolinium-based contrast agent (Magnevist Bayer HealthCare, Pittsburgh, PA) was used in experimental mice at a dose of 0.1 mm/kg, diluted in gadopentetate dimeglumine (140 ul total volume), and was administered at 20 ul/sec via tail vein catheter injection with a 50 µl dead space in the line filled of saline.

### Human subjects

TMEM-MRI activity was measured in breast cancer patients. This study was designed by Montefiore-Einstein cancer center investigators, approved by the Albert Einstein Institutional Review Board, and conducted in accordance with the ethical principles derived from international guidelines, including the International Council for Harmonisation Good Clinical Practice guidelines, the Declaration of Helsinki, and local regulations on the conduct of clinical research. All the participants provided written informed consent before enrollment. The inclusion criteria were the following: breast mass >1 cm with biopsy-proven histology of invasive breast carcinoma (any histologic type and ER, PR, HER2 status), age ≥18 years, ECOG performance status 0–1, ability to undergo MRI with gadolinium enhancement, no known or suspected renal impairment, normal organ and marrow function, weight less than or equal to the MRI table limit, ability to understand and willingness to sign a written informed consent. The exclusion criteria were the following: prior chemotherapy of radiation therapy to the ipsilateral breast, breast prosthetic implants (silicone or saline), use of any investigational agent within 30 days of starting the study, uncontrolled intercurrent illness including, but not limited to, ongoing or active infection, symptomatic congestive heart failure, unstable angina pectoris, cardiac arrhythmia, or psychiatric illness/social situations that would limit compliance with study, pregnancy, and lactation. The clinical and pathological characteristics of the tumors are summarized in Fig. 5h.

### Development of the mouse TMEM activity-MRI assay

The MRI images were obtained on a 9.4 Tesla Agilent Direct Drive MRI/MRS system. Images were acquired with a 24 mm diameter surface receive-only coil within a 12 cm diameter pin-switch driven volume transmit coil. High-resolution T2-weighted images were used to determine the placement of the two slices in the permeability estimation, one for the arterial source and one for the tumor slice. The GRE images were collected with a field of view of 23 mm^2^, matrix size of 96 × 128, thickness of 1 mm, image repetition time (TR) of 23 ms, echo time (TE) of 3 ms, flip angle (FA) was 28 degrees, acquisition bandwidth of 100 kHz, for an image temporal resolution of 2 s (signal averages = 1) or 4 s (SA = 2). The samples in the dynamic study were sampled every 4 s and for 10 min, with the contrast (gadolinium) being injected at 1 min. Prior to dynamic image acquisition, a T1 measurement was completed by varying the FA between 2 and 60 ms, to which T1 was fit on a pixel-by-pixel basis, and the low FA data (FA 2, 4, 6, and 8 degrees) were used along with the dynamic data to fit dynamic T1 to the dynamic curve pixel-by-pixel for determination of CA concentration.

During a contrast-enhanced MRI exam, a gadolinium-based contrast agent (GBCA) is typically injected and CA passage into the tumor tissue is visualized by comparing image intensity changes pre- and post-contrast (subtraction-based contrast)^89^. Alternatively, high temporal resolution MRI of the dynamic passage of the GBCA through tissue beds using longitudinal (T1) relaxation-based agents can be used to measure tissue permeability to the GBCA. Typically, these contrast agents are T1-based agents, for which the change in tissue relaxivity associated with the contrast agent’s presence can be used to approximate the agent’s blood and tissue time-dependent concentration. Mathematical deconvolution methods can be used to extract the tissue response function from the tissue signal response function using the measured or inferred arterial input concentration function of the contrast agent, the form of which is derived from the differential equation:

1 $$V_e\frac{{dC_e(t)}}{{dt}} = PS\rho \left[ {C_p\left( t \right) - C_e(t)} \right],$$

where C_p_(t) and C_e_(t) are the GBCA concentration in the blood plasma and tissue, respectively, P is the GBCA endothelial permeability, S is the capillary wall surface area, and ρ is the tissue density.

The product PS is often called the permeability-surface area product, which assumes that delivery of the GBCA and perfusion are sufficient to ensure that the permeability is the dominant determinant of GBCA exflux into the tissue. Endothelial permeability of the GBCA is usually represented as the permeability transfer constant, or k_trans_;

2 $$K_{{\mathrm{trans}}} = PS\rho$$

If we use the convention that the concentration of GBCA in the imaging voxel, Ct(t) is given by the volume weighted concentrations of the plasma *V*_*p*_ and tissue *V*_*e*_;

3 $$C_t\left( t \right) = V_pC_p\left( t \right) + V_eC_e(t)$$

Solution of the differential leads to a convolution integral, which can be simplified by assuming that the uptake of GBCA into the extravascular/extracellular space is minimal during the measurement. Because the entire measurement process outlined above is limited to the ‘first pass’ of the GBCA, this assumption is valid and simplifies the convolution integral to the following:

4 $$C_t\left( t \right) = k_{{{{\mathrm{fp}}}}}\int _0^t C_p\left( {t^\prime } \right)dt^\prime$$

which shows that the Gd concentration in the tissue is modeled as a convolution of the tissue’s permeability-surface product (*k*_fp_) and the integrated GBCA delivery.

This estimation is valid during the first pass of the contrast bolas, before the recirculation of the agent. The use of this first-pass leakage profile has been shown to give a more accurate estimation of the TMEM-associated vascular opening (TAVO, also shown as *k*_fp_) compared to the Multicompartmental Model^30^.

A baseline T1 map needs to be estimated to allow for the calculation of a concentration map in real-time. Multiple GRE images with varying flip angles (FA) are acquired and Eq. 5, based on the Ernst formula, is fit to give a T1 estimate:

5 $${\it{s}} = {\it{m}}_0\left( {{{{\mathrm{sin}}}}{\it{{\mathrm{FA}}}}} \right)\frac{{1 - {\it{e}}^{ - {\it{{\mathrm{TR}}}}/{\it{T}}1_0}}}{{1 - \left( {{{{\mathrm{cos}}}}{\it{{\mathrm{FA}}}}} \right) \ast {\it{e}}^{ - {\it{{\mathrm{TR}}}}/{\it{T}}1_0}}}$$

To fit this model for T1_0_, FA = (2, 4, 6, 8, 10, 12, 16, 20, 25, 30, 40) degrees were acquired, with TR = 15 ms and TE = 3.2 ms. Levenburg–Marquart nonlinear least squares algorithm is used to fit Eq. 5. This gives a baseline T1_0_ estimate.

After gaining the baseline T1_0_ map, the GBCA is injected and the dynamic GRE series (S(t)) of images is collected, with FA = 25 degrees sampled every 4 s. A dynamic T1 series (T1(t)) is estimated directly using the following:

6 $$\frac{1}{{T1\left( t \right)}} = \frac{{ - 1}}{{{\mathrm{TR}}}} \ast ln\left( {\frac{{1 - D}}{{1 - \left( {\cos {\mathrm{FA}}} \right) \ast D}}} \right)$$

7 $$D = \frac{{S\left( t \right) - S(0)}}{{m_0{{{\mathrm{sin}}}}{\mathrm{FA}}}} + \frac{{1 - e^{ - {\it{{\mathrm{TR}}}}/{\it{T}}1_0}}}{{1 - \left( {{{{\mathrm{cos}}}}{\mathrm{FA}}} \right) \ast e^{ - {\it{{\mathrm{TR}}}}/{\it{T}}1_0}}}$$

The Gd concentration map (C(t)) is then calculated with the following:

8 $$C\left( t \right) = \frac{{\frac{1}{{T1\left( t \right)}} - \frac{1}{{T1_0}}}}{{R1}}$$

where the relaxivity of the Gd is R1 = 3.2 s^−1^ mM^−1^.

To narrow our analysis on vascular leakage elicited by TAVO events, an arterial source must first be found. So, we acquire two slices in our scans, one through the tumor (C_t_) and one through the artery (C_a_). Using Eq. 4 with C_p_ = C_a_, weighted least squares is used to estimate *k*_fp_ with higher weights given to the points at the top of the C_a_ curve.

To graphically compare the TMEM-Associated Vascular Opening (TAVO) events of mouse breast carcinomas among different conditions, we calculated the ratio of values above a permeability threshold in the entire histogram (Eq. 9). The optimal threshold for class separation (early versus late carcinomas in PyMT mice) was found to be ~0.001. In the following equation (Eq. 9), *H*_i_ represents the histogram of *k*_fp_ values found within the tumor.

9 $${\mathrm{TMEM}}\;{\mathrm{Activity}} - {\mathrm{MRI}} = \mathop {\sum }\limits_{i = th}^{.04} H_i/\mathop {\sum }\limits_{i = 0}^{.04} H_i$$

### Development of the human TMEM activity-MRI assay

TMEM activity was measured in a small preliminary study of breast cancer patients after consent in accord with an approved IRB protocol. TMEM activity was measured on a Philips 3 T Ingenia Elition. Dynamic contrast-enhanced (DCE) imaging was conducted following intravenous administration of Gadoterate (0.1 mm/kg) administered as a bolus a power injector. The dynamic contrast-enhanced MRI (DCE-MRI) protocol used for clinical assessment was modified to permit the collection of a T1 relaxometry data set prior to CA injection and a dynamic data set collected during the CA injection. The DCE-MRI data set began 1 min prior to injection and continued for at least 90 s following injection using a 4D-TRAK XD imaging protocol which employed compressed sense (factor 8) and both keyhole (20%) and half scan (factors 0.625 and 0.878) parallel imaging providing rapid (3 to 5 s) volume acquisitions. Image volumes were acquired in rapid succession, with a high-resolution matrix (1 mm^3^ isotropic or nearly isotropic voxels), and (typically) a TR of 3.9 ms, TE 1.95 ms, temporal 3D volume resolution of 3.2 to 4.6 s, flip angle 28 degrees, and between 60 and 120 sequential 3D volumes. Imaging covered both breasts, as well as the heart, from which an arterial signature of CA uptake was acquired. Pre-contrast and post-contrast conventional 3D images were acquired after the dynamic images, allowing delineation of the tumor volume. Fitting of the convolution equation followed that described for the animal data, with a similar calculation of the TMEM Activity-MRI.

### Histology (H&E) and immunohistochemistry for IBA1 and TMEM doorways

After mice were sacrificed, all mammary tumors were extracted and immersed in 10% formalin in a volume ratio of tumor to formalin of 1:7. Tissues were fixed for 24 to 48 h and embedded in paraffin, then processed for histological examination. One 5 µm section from each tumor was stained for hematoxylin and eosin (H&E) and one for TMEM. The TMEM doorway assay is a triple-stain IHC for predicting metastatic risk, in which three antibodies are applied sequentially and developed separately with different chromogens on a Dako Autostainer. TMEM doorway stain was performed as previously described^2^, except that in this study we used anti-panMena antibody (510693; BD Biosciences) to detect Mena-expressing cancer cells. To visualize macrophages, we used anti-IBA1 antibody (019-19741; Wako) for mouse and anti-CD68 (MO876; Dako) for human tumors. To visualize endothelial cells, we used anti-endomucin (SC-65495; Santa Cruz) for mouse and anti-CD31 (MO823; Dako) for human tumors. Appropriate areas containing invasive cancer tissue suitable for TMEM doorway analysis were identified by low-power scanning using the following criteria: high density of tumor, adequacy of a tumor, lack of necrosis or inflammation, and lack of artifacts such as retraction or folds. TMEM doorway scoring was performed as previously described^2,36^. The assessment of TMEM doorway scores was performed with Adobe Photoshop on ten high-power (400X) digital images of the most representative areas of the tumor. The total number of TMEM doorways for each image were tabulated, and the scores from all ten images were summed to give a final TMEM doorway density for each patient sample, expressed as the number of TMEM doorways per total area (ten high-power [400X] fields). A representative high-power magnification image showing the tripartite TMEM doorway is indicated in Supplementary fig. 3a. IBA1 single immunohistochemistry was performed with the IBA1 primary antibody used in the TMEM immunohistochemistry, and a representative high-power magnification image is indicated in Supplementary fig. 3b.

### Multichannel immunofluorescence

For all multichannel immunofluorescence experiments, slides were first deparaffinized by melting at 60 ^o^C in an oven equipped with a fan for 20 min, followed by 2X xylene treatment for 20 min. Slides were then rehydrated, and antigen retrieval was performed in 1 mM EDTA (pH 8.0) at 97^o^ C for 20 min in a conventional steamer, followed by incubation in a blocking buffer solution (10% FBS, 1% BSA, 0.0025% fish skin gelatin in 0.05% PBST) for 60 min at room temperature. Specific considerations were then considered depending on the assay performed, as described below.

#### MENA^INV^ immunofluorescence

After standard slide preparation as described above, slides were incubated with chicken anti-MENA^INV^ (0.25 μg/mL, generated in the lab of Dr. John S. Condeelis) in a blocking buffer for 60 min at room temperature. Samples were washed three times in 0.5% PBST and incubated with an HRP-conjugated IgG anti-chicken secondary antibody for 60 min at room temperature. After washing, slides were incubated with biotinylated tyramide (Perkin Elmer; Opal 4-color Fluorescent IHC kit) diluted at 1:50 in amplification buffer for 10 min. After washing, slides were incubated with spectral DAPI for 5 min and mounted with ProLong Gold antifade reagent (Life Technologies). The slides were imaged on the Pannoramic 250 Flash II digital whole slide scanner, using a 20 × 0.75NA objective lens. Tissue suitable for scanning was automatically detected using intensity thresholding.

#### TMEM activity-dextran assay

Assessment of TMEM-mediated vascular opening (TAVO) was performed using multichannel-IF in an FFPE section with a sequential TMEM triple-IHC section already stained. Each slide was stained with a primary antibody mixture cocktail against rat anti-endomucin (1:500; sc-65495; Santa Cruz) and rabbit anti-TMR (1:1,000; A-6397; Life Technologies). Slides were then washed three times in 0.05% PBST, and incubated with a secondary antibody mixture cocktail, including donkey anti-rabbit Alexa-488 and goat anti-rat Alexa-568, both at 1:200 dilution for 60 min at room temperature. After washing (0.05% PBST 3X), slides were incubated with spectral DAPI for 5 min and mounted with ProLong Gold antifade reagent (Life Technologies). The slides were imaged on the Pannoramic 250 Flash II digital whole slide scanner, using a 20 × 0.75NA objective lens. Tissue suitable for scanning was automatically detected using intensity thresholding. Whole tissue images were uploaded in Pannoramic Viewer version 1.15.4 (3DHISTECH). To investigate whether highly permeable blood vessels were associated with TMEM doorways, multiple 40X fields were captured to obtain ~20–25 vascular profiles for each case. A “vascular profile” was defined as an endomucin^+^ blood vessel with clear margins, either longitudinally or in cross-section. Vessels in the vicinity or continuing away from the field of view were excluded from this analysis because it was not possible to access the entire perivascular area associated with them. In each image, the endomucin and dextran-TMR channels were each thresholded just above the background based upon intensity by using contrast adjustment. For each vascular profile, the endomucin channel was then used as an exclusion mask to the dextran channel to directly designate an ROI that belonged exclusively to the extravascular portion of the dextran. Because IF staining may result in a certain number of nonspecific “speckles” with a positive TMR signal, we used an arbitrary threshold of >20 pixels around a vascular profile to consider it a “leaky vascular profile”. The sequential TMEM IHC sections were then used to assess whether these leaky profiles had an associated TMEM structure. To directly compare TAVO-dependent dextran leakage among different groups of mice, the extravascular dextran ROI was expressed in each image as an area fraction, and an average among all ROIs for each mouse was reported.

#### Quantification of disseminated tumor cells (DTCs) in mouse lungs

The assessment of DTCs in the lungs was performed in the MMTV-PyMT mouse model, based on the availability of commercial anti-PyMT antibodies for specific detection of PyMT-expressing tumor cells, indicative of this mouse model of breast carcinoma. Each lung section was stained with primary antibody mixture cocktail against mouse anti-pancytokeratin (PanCK; 1:1,000; C2562; Sigma) and rat anti-PyMT (PyMT; 1:400; NB100-2749; Nobus Biologicals). Slides were then washed three times in 0.05% PBST and incubated with a secondary antibody mixture cocktail, including goat anti-mouse Alexa-488 and donkey anti-rat Alexa-568, both at 1:200 dilution for 60 min at room temperature. After washing (0.05% PBST 3X), slides were incubated with spectral DAPI for 5 min and mounted with ProLong Gold antifade reagent (Life Technologies). The slides were imaged on the Pannoramic 250 Flash II digital whole slide scanner, using a 20 × 0.75NA objective lens. Tissue suitable for scanning was automatically detected using intensity thresholding. Whole tissue images were uploaded in Pannoramic Viewer version 1.15.4 (3DHISTECH). DTCs were identified as single PanCK^+^PyMT^+^DAPI^+^ cells and expressed as mean (i.e., DTCs per high-power field) for each mouse.

### Intravasation (circulating tumor cell) assay

The in vivo intravasation assay was performed as previously described^5,39,90,91^. Mice were anesthetized with isoflurane, and blood was taken from the right ventricle by heart puncture, using 25 G needles coated with heparin. Erythrocytes were lysed using 10 ml of 1X RBC lysis buffer (multi-species, eBioscience). The samples were centrifuged at 200 × *g* for 5 min, then cell pellets were reconstituted in 10 ml of Dulbecco’s modified Eagle medium (DMEM/F-12) supplemented with 20% fetal bovine serum (FBS) and plated in 10-cm Petri dishes. After cells were attached, single tumor cells were counted. The total number of cells counted was divided by the volume of blood taken. Tumor cells counted were either CFP- or Dendra2-positive, thus confirming their identity as tumor cells. As a negative control, blood from a non-tumor-bearing mouse was analyzed, and the complete absence of epithelial cells was confirmed.

### Statistical analysis

All statistical analyses, including graph/plot generation and statistical hypothesis testing, were performed in GraphPad Prism 8 software. The variables in our study (TMEM score, TMEM Activity-MRI, TMEM Activity-Dextran, circulating tumor cells per mL blood, disseminated tumor cells) were all continuous and, as such, presented as dot-plots with means and their corresponding Standard Deviation (SD). Statistical comparisons between two independent groups were performed using a two-tailed Mann–Whitney *U*-test, and those between more than two independent groups were performed using Kruskal–Wallis one-way analysis of variance. For longitudinal analyses, repeated-measures ANOVA was used. Statistical correlations were performed by Spearman’s correlation, with the data presented as scatterplots with fit lines and their corresponding *P* values. Mouse studies were conducted in at least three independent animal cohorts, which were pooled together for statistical analysis.

Sample size calculations for all the validation studies were performed using the following parameters: significance level (adjusted for sidedness) of 0.025, probability of type II error of 0.2 (i.e., statistical power 0.8), and expected difference in means equal to 1.5 SD units, based on the assumption that the SD of the response variable was 1 unit. Based on the above, a total of 18 animals was calculated to enter the study. The experiments were initially designed to include >18 animals in each study, to account for mice that died during an MRI session, mice that fitted one or more exclusion criteria, and finally for statistical outliers. The following animals were removed from the study based on exclusion criteria: Mice whose tumors were overly necrotic or cystic; mice with inadequate tumor tissue to perform IHC or IF; mice with less than 0.5 ml of blood collected through heart puncture for the CTC assay; and mice that were either obese (>35 g), or emaciated (<17 g), upon reaching the experimental endpoints. As such, slight deviations can be observed in the total number of mice in each individual figure panel, accounting for the exclusion criteria applied in each case.

Transgenic MMTV-PyMT animals, as well as animals transplanted with patient-derived tumors (e.g., HT17 xenografts), were housed in cages of five animals per the regulations of the Albert Einstein College of Medicine (AECOM) Animal Care and Use Committee. Once the mice reached the criteria for inclusion into the experimental pipeline (tumors with a diameter of ~2–3 mm), they were randomly allocated to rebastinib-, chemotherapy-, or vehicle-treated groups.

The two pathologists (M.H.O. and J.G.J.) involved in TMEM scoring were blinded to the specific group allocations, as were all the scientists performing CTC scoring, all IF/IHC analyses, and all MRI feature quantifications. Importantly, the TMEM Activity-MRI maps, upon which TMEM-MRI-Activity is determined, was conducted prior to and without knowledge of the TMEM pathology score (and scored by different individuals) and therefore was blinded.

## Supplementary information

Supplementary Material

## Data Availability

The original contributions presented in the study are included in the article and supplementary material. Further inquiries can be directed to the corresponding authors. No datasets were generated or analyzed during the current study.
