# Supplementary material for: Assessment of MRI to estimate metastatic dissemination risk and prometastatic effects of chemotherapy
Source: NPJ Breast Cancer. 2022 Sep 2;8:101. doi: 10.1038/s41523-022-00463-5 (PMC9440218; doi:10.1038/s41523-022-00463-5)
Supplement: Supplementary file 1 — Supplementary Material [file 41523_2022_463_MOESM1_ESM.pdf]

Supplementary Figure 1

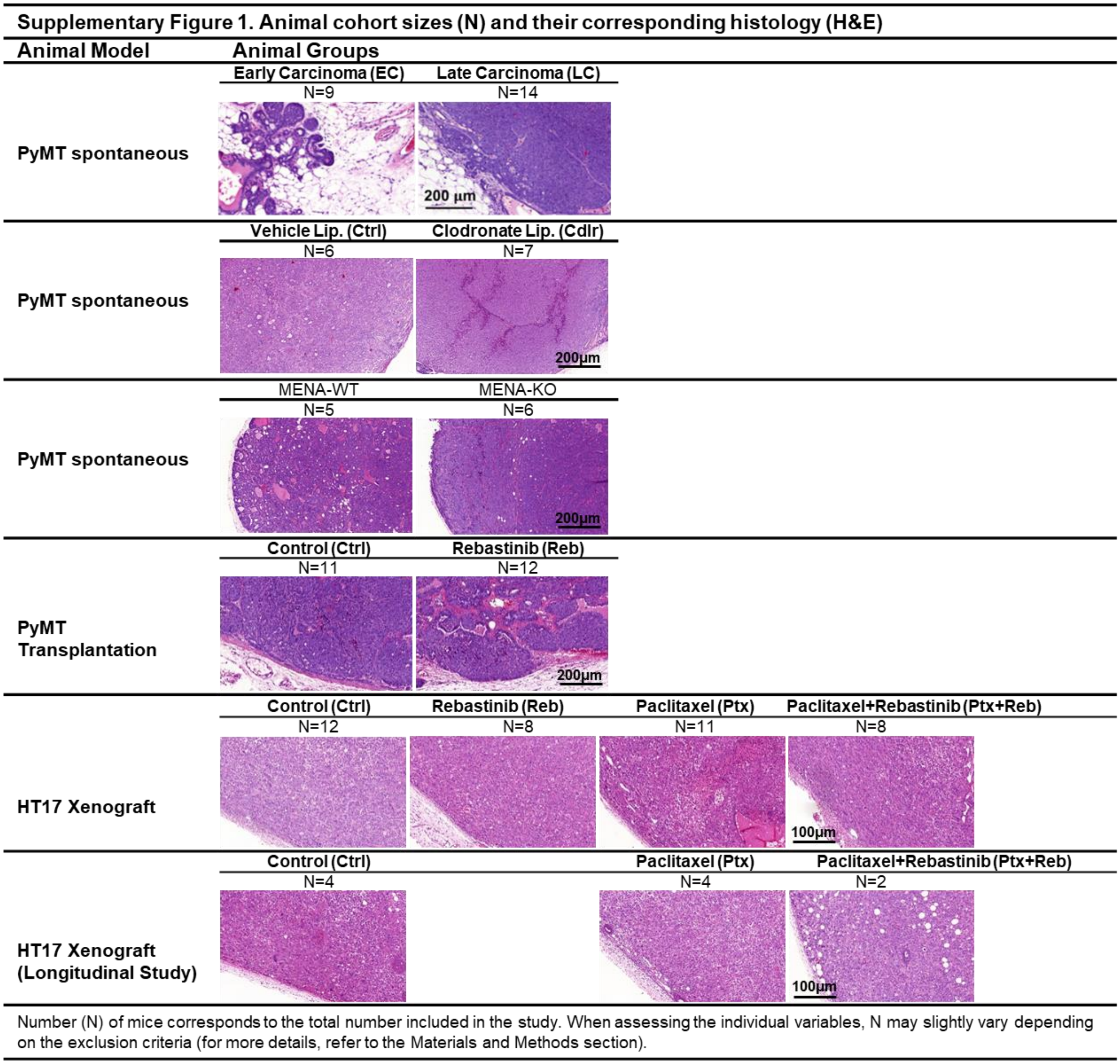

Supplementary Figure 1. Animal cohorts used in the current study. Each row provides specific information on the experimental and control groups for each model, the cohort size (N), and corresponding, representative histology (H&E) in low magnification.

Supplementary Figure 2

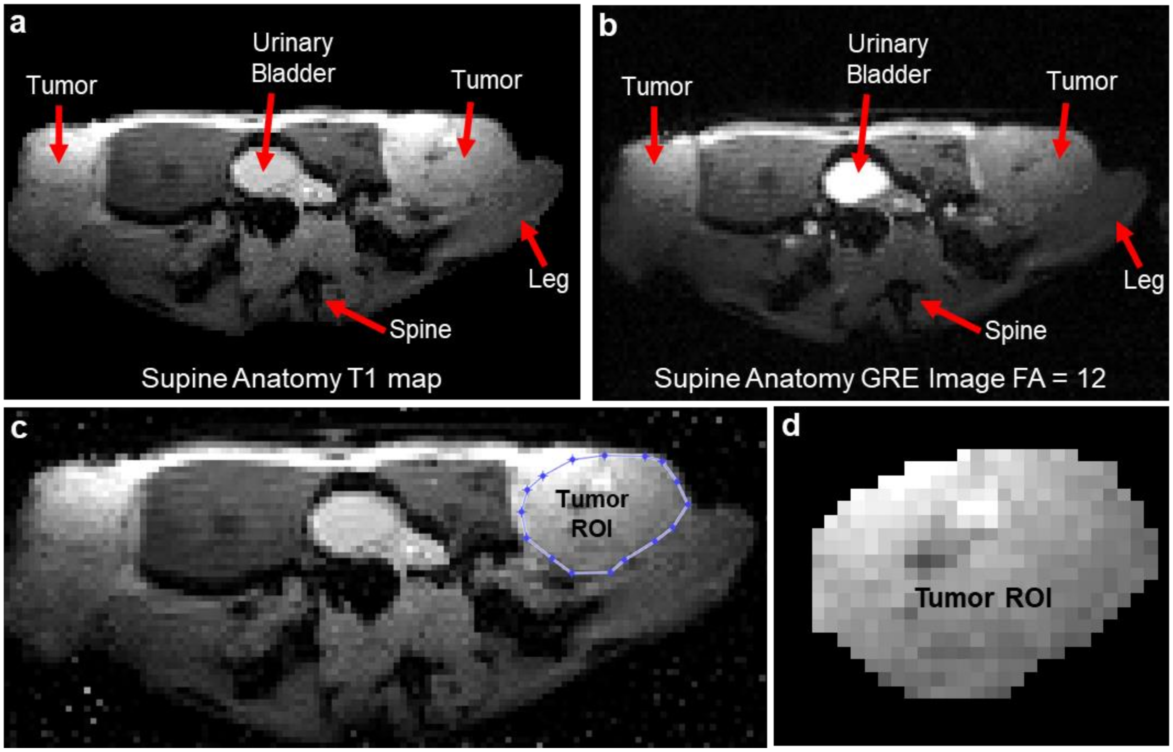

Supplementary Figure 2. Selection of tumor region of interest (Tumor ROI) using anatomical overviews in Magnetic Resonance Imaging (MRI). (a) Anatomical landmarks for the collected images given a T1 map image. The T1map is not used for drawing the ROIs, but it gives out baseline for calculating the concentration maps. **(b)** The GRE Image with a FA = 12 is used to draw the ROI of the tumor. The FA of 12 has the highest contrast due to it being the FA with the maximal signal response (shown in main Figure 1a). **(c)** Example to tumor ROI extraction from the GRE image and superimposed on the T1map. **(d)** T1 map of the extracted tumor.

Supplementary Figure 3

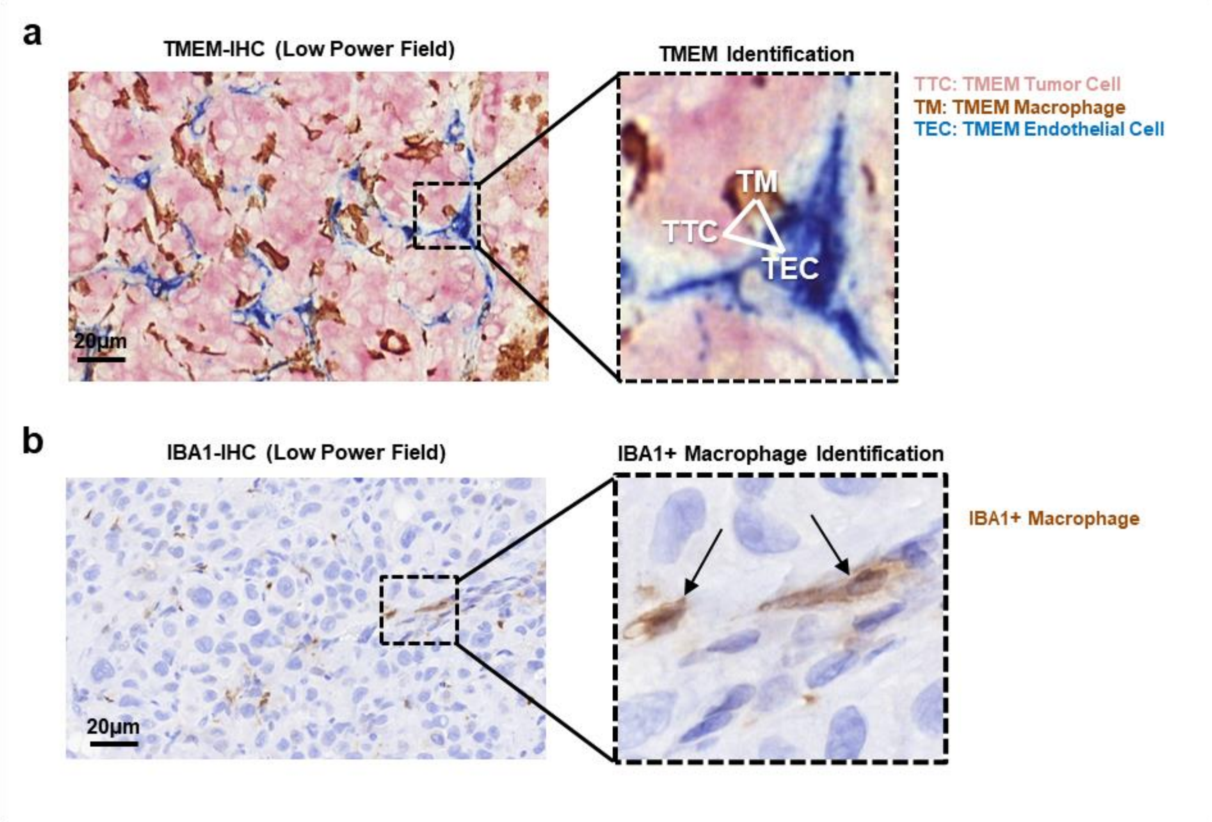

Supplementary Figure 3. High Resolution images of IBA1 and TMEM immunohistochemistry. (a) Triple-stain immunohistochemistry for TMEM doorway identification and quantification, showing in white triangle the three cell components of each TMEM doorway: TMEM macrophage (TM), TMEM endothelial cell (TEC), and Mena expressing TMEM tumor cell (TTC). Note that all three different cell types must be in direct physical proximity to one another to define this tripartite structure as “TMEM doorway”. **(b)** IBA1+ Macrophage immunohistochemistry. The arrows point to macrophages in the breast tumor microenvironment.

Supplementary Figure 4

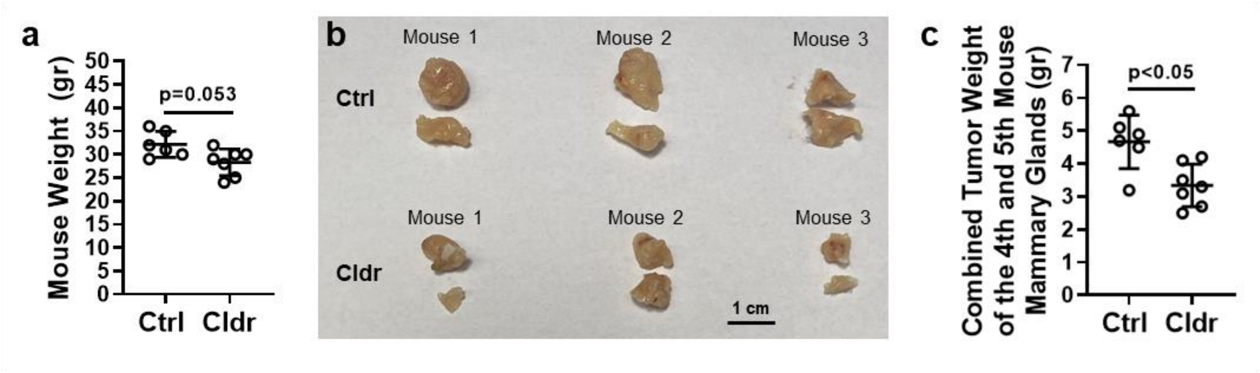

Supplementary Figure 4. Tumor burden of MMTV-PyMT mice treated with either PBS- or clodronate-liposomes. (a) Weight (gr) of MMTV-PyMT mice treated with either PBS- or clodronate-liposomes at the experimental endpoint. Mann Whitney U-test. **(b)** Representative tumor burden (all tumors from 4th and 5th mammary gland combined) from 1 of the 2 mouse cohorts treated with either PBS- or clodronate-liposomes. **(c)** Weight (gr) of all combined tumors excised from the 4th and 5th mammary glands of MMTV-PyMT mice treated with either PBS- or clodronate-liposomes. Mann Whitney U-test. Ctrl, control; Cldr, clodronate.

Supplementary Figure 5

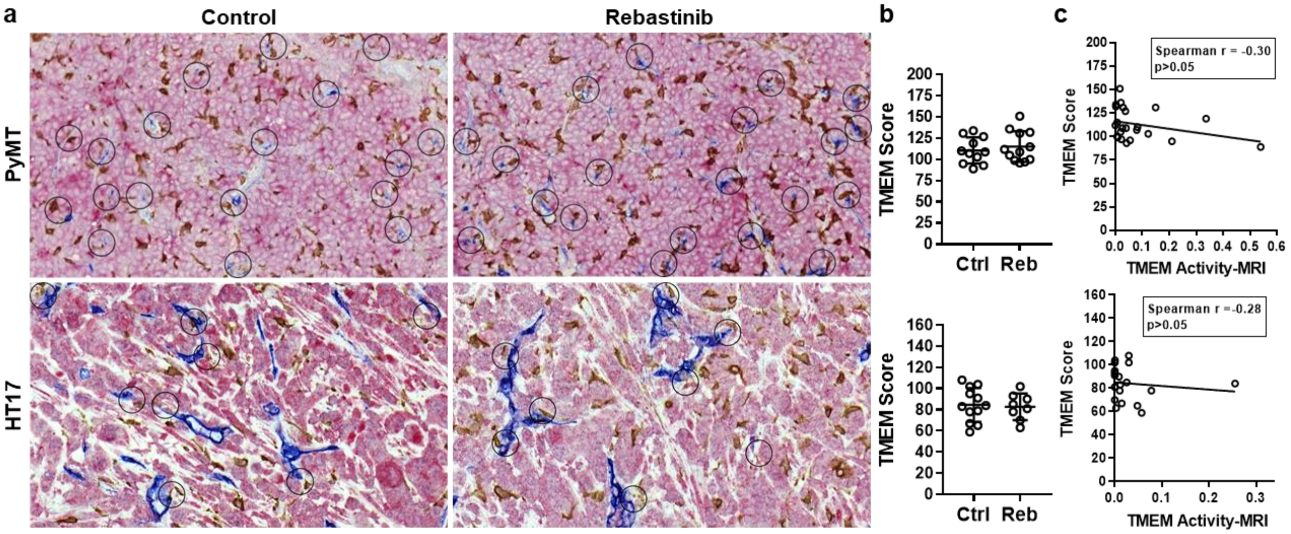

Supplementary Figure 5. TMEM doorway analysis in MMTV-PyMT and HT17 mice treated with rebastinib or vehicle control. (a) TMEM identification by triple-stain IHC and representative images from vehicle-treated (left column) and rebastinib-treated (right column) breast carcinomas in MMTV-PyMT (upper low) and HT17 (bottom row) mice. Magnification 40X. **(b)** Quantification of TMEM doorways (TMEM score), assessed in 10 high-power fields (HPFs), in MMTV-PyMT (upper graph) and HT17 (bottom graph) mice. Mann-Whitney U test. **(c)** Correlation between TMEM score (as quantified in B) and TMEM Activity-MRI (as quantified in main Figure 2c-c') in MMTV-PyMT (upper graph) and HT17 (bottom graph) mice treated with rebastinib. Spearman's rank correlation coefficient.

Supplementary Figure 6

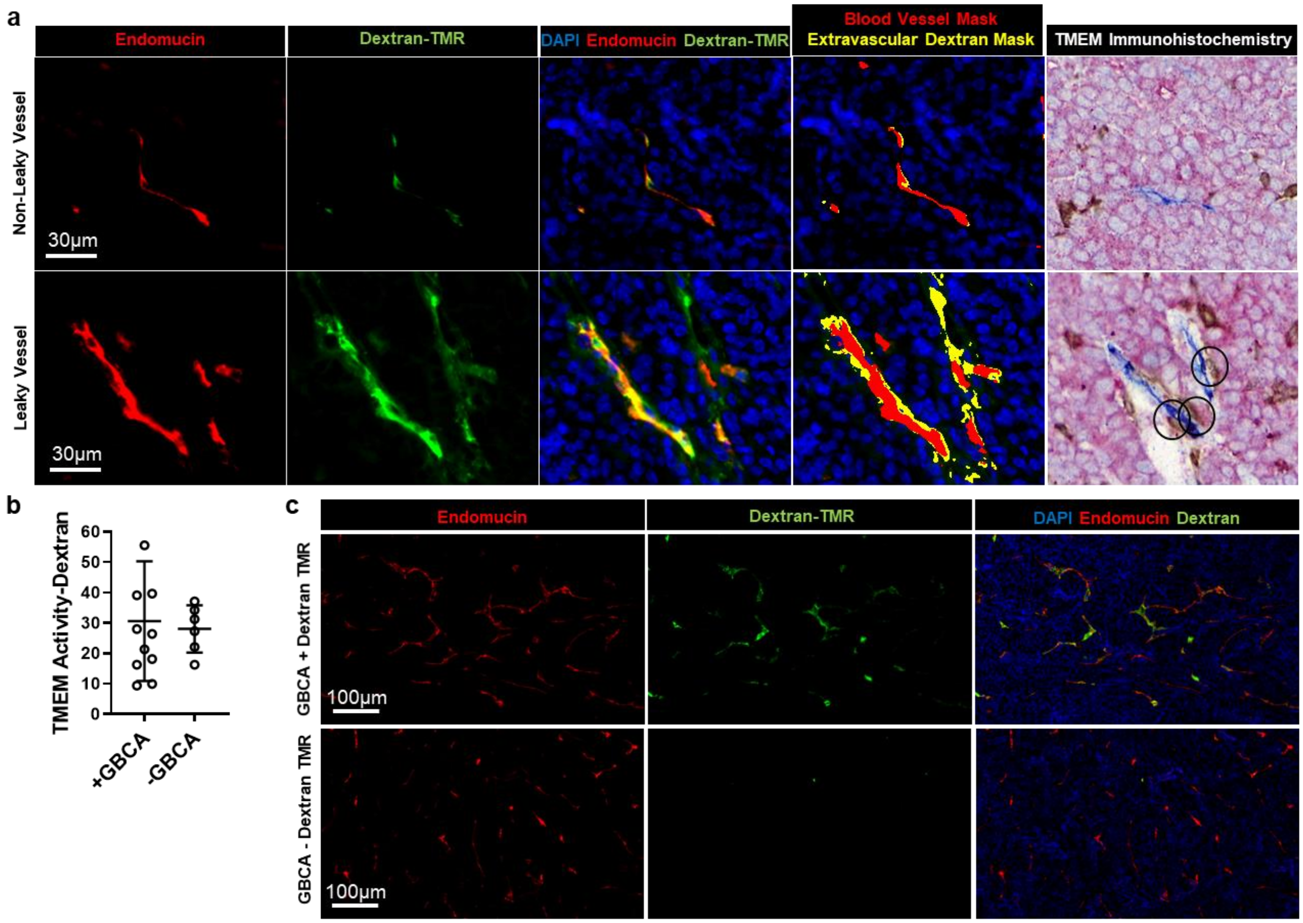

Supplementary Figure 6. Development of an adapted TMEM Activity-Dextran protocol from previously published TMEM Activity-Dextran assay (Karagiannis et al. JoVE, 2018). Because the published TMEM Activity-Dextran assay did not include assessment of extravascular dextran following administration of gadolinium-based contrast agent (GBCA), a series of control assays were conducted to validate this assay in conjunction with MRI studies. **(a)** Multichannel IF of endomucin (first column), dextran-TMR (second column), their merged image along with DAPI (third column), the corresponding thresholded blood vessel and extravascular dextran masks (fourth column), and the corresponding sequential section of TMEM IHC (fifth column) in MMTV-PyMT mice that received GBCA for MRI studies prior to Dextran-TMR injection. Upper row: Vascular profile without TMEM doorways, presented as a non-leaky profile. Bottom row: TMEM-associated vascular profile, presented as a leaky profile. Scale bar, 30um. **(b)** Quantification of TMEM-mediated vascular opening (TMEM activity), as assessed via the TMEM Activity-Dextran assay in PyMT mice, either subjected to MRI studies (+GBCA group; N=10) or not subjected to MRI studies (-GBCA group; N=6). Note that +GBCA mice are the control (non-treated with rebastinib) mice presented in main Figure 2, whereas –GBCA mice are a matched control for this subgroup. Comparison of TMEM Activity-Dextran reveals similar levels of TMEM-mediated vascular opening in the two groups, further suggesting that GBCA does not affect TMEM-dependent vascular opening. **(c)** GBCA interference control. PyMT mice received GBCA plus (N=3) or minus (N=3) Dextran-TMR, to assess if GBCA gives false-positive dextran signal. As shown in the bottom row, mice injected with GBCA alone did not show any positive dextran signal. However, mice that received Dextran-TMR following GBCA administration (upper row) demonstrated positive dextran signal. The GBCA interference control confirms that GBCA administration does not give false-positive dextran signal. Images are representative of three mice for each condition.
